# The Response of Carbon Uptake to Soil Moisture Stress: Adaptation to Climatic Aridity

**DOI:** 10.1111/gcb.70098

**Published:** 2025-03-10

**Authors:** Giulia Mengoli, Sandy P. Harrison, I. Colin Prentice

**Affiliations:** ^1^ Georgina Mace Centre for the Living Planet, Department of Life Sciences Imperial College London Ascot UK; ^2^ Department of Geography and Environmental Science, School of Archaeology, Geography and Environmental Science (SAGES) University of Reading Reading UK; ^3^ Ministry of Education Key Laboratory for Earth System Modelling, Department of Earth System Science Tsinghua University Beijing China

**Keywords:** adaptation, aridity, critical thresholds, light‐use efficiency, optimal water use, P model, primary production, soil‐moisture stress

## Abstract

The coupling between carbon uptake and water loss through stomata implies that gross primary production (GPP) can be limited by soil water availability through reduced leaf area and/or stomatal conductance. Ecosystem and land‐surface models commonly assume that GPP is highest under well‐watered conditions and apply a stress function to reduce GPP as soil moisture declines. Optimality considerations, however, suggest that the stress function should depend on climatic aridity: ecosystems adapted to more arid climates should use water more conservatively when soil moisture is high, but maintain unchanged GPP down to a lower critical soil‐moisture threshold. We use eddy‐covariance flux data to test this hypothesis. We investigate how the light‐use efficiency (LUE) of GPP depends on soil moisture across ecosystems representing a wide range of climatic aridity. ‘Well‐watered’ GPP is estimated using the sub‐daily P model, a first‐principles LUE model driven by atmospheric data and remotely sensed vegetation cover. Breakpoint regression is used to relate daily β(θ) (the ratio of flux data–derived GPP to modelled well‐watered GPP) to soil moisture estimated via a generic water balance model. The resulting piecewise function describing β(θ) varies with aridity, as hypothesised. Unstressed LUE, even when soil moisture is high, declines with increasing aridity index (AI). So does the critical soil‐moisture threshold. Moreover, for any AI value, there exists a soil moisture level at which β(θ) is maximised. This level declines as AI increases. This behaviour is captured by universal non‐linear functions relating both unstressed LUE and the critical soil‐moisture threshold to AI. Applying these aridity‐based functions to predict the site‐level response of LUE to soil moisture substantially improves GPP simulation under both water‐stressed and unstressed conditions, suggesting a route towards a robust, universal model representation of the effects of low soil moisture on leaf‐level photosynthesis.

## Graphical abstract

Written Summary: Plants need water to grow, but plants in arid climates generally use water more conservatively even in wet conditions and adjust their carbon uptake as soil moisture declines. We use flux data from individual sites to develop a new empirical function that predicts how maximum plant productivity is affected by increasing aridity and how productivity declines under subsequent water stress. This approach offers a universal way to account for water stress in ecosystem and climate models and should improve our ability to model how vegetation will respond to future climate change.

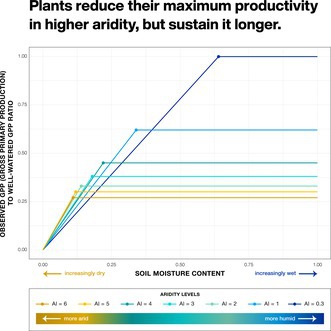

## 1 Introduction

The tight coupling between carbon uptake and water loss via stomata (Cowan and Farquhar 1977; Manzoni et al. 2011a) implies that gross primary production (GPP) can be limited by water availability through reduced vegetation cover and leaf area index, reduced stomatal conductance, or a combination of these. Reduced evapotranspiration under water stress causes increased sensible heat flux, warming the atmosphere above the canopy, which, in turn, causes a further reduction in transpiration and plant carbon uptake (Gentine et al. 2016; Grossiord et al. 2020; Seneviratne et al. 2010). Plants need to coordinate these two processes—water loss and carbon uptake—to maximise assimilation while minimising water loss. Although this trade‐off is well established as a general concept, determining whether the reduction in GPP is due to stomatal or non‐stomatal limitation or both remains challenging; moreover, the mechanisms behind non‐stomatal limitation are not entirely clear. However, plants in seasonally dry environments evidently have to deal with low soil moisture and must adjust to it by reducing either leaf area or photosynthesis. In this study, we isolate the effects of soil moisture on the light‐use efficiency (LUE) of photosynthesis relative to the well‐watered condition, which we represent using the sub‐daily version of the P model (Mengoli et al. 2022) using remotely sensed data on fractional absorbed solar radiation (fAPAR), a measure of green vegetation cover, as an input to the model.

There is evidence that soil moisture, rather than atmospheric demand, is the principal immediate constraint on GPP in arid and semi‐arid ecosystems (Dubey and Ghosh 2023; Pei et al. 2020; Xu et al. 2023, but see also Verma and Ghosh 2024, who showed how *antecedent* vapour pressure deficit (VPD) acts on GPP via its effect on soil moisture). GPP is substantially reduced—much more than total ecosystem respiration—in response to drought (e.g., Shi et al. 2014). Liu et al. (2020) showed that soil moisture is the dominant water stress on vegetation over 70% of the global land area. However, the response of GPP to water stress in models from the previous round of the Coupled Model Intercomparison Project, CMIP5, is too strong (Huang et al. 2016) and representation of the soil moisture effects on GPP remains one of the largest sources of uncertainty in carbon cycle models (Trugman et al. 2018). Many studies have focused on the impact of drought on vegetation greenness (e.g., Li et al. 2023); but soil moisture stress also impacts light‐use efficiency (LUE) directly, which further reduces GPP (Lv et al. 2023; Xing et al. 2023). Thus, it is important to account for the impact of soil moisture stress on LUE, as well as on vegetation greenness.

The P model is a model for the LUE of GPP based on eco‐evolutionary optimality (EEO) theory (Cai and Prentice 2020; Stocker et al. 2020; Wang et al. 2017). It captures the trade‐off between CO_2_ uptake and water loss and provides realistic estimates of the seasonal and diurnal cycles of GPP under well‐watered conditions, performing as well as or better than more complex models despite having far fewer parameters (Harrison et al. 2021; Stocker et al. 2020). The sub‐daily version of the P model, tested at multiple sites representing different biomes, climates and vegetation types, accurately simulates diurnal and seasonal cycles of GPP in well‐watered climates without requiring any plant functional type‐specific parameters (Mengoli et al. 2022). But despite the model's accuracy as judged against GPP inferred from eddy‐covariance flux measurements in well‐watered ecosystems, it overestimates GPP in seasonally dry environments. This is because although the model accounts for the effect of VPD in reducing stomatal conductance, it does not account for any additional impact of soil‐moisture stress. Given the potential for EEO‐based models to provide robust representations of vegetation and land‐surface exchanges with the atmosphere (Franklin et al. 2020; Harrison et al. 2021; Mengoli et al. 2022), it is important to develop a well‐founded approach to implement soil‐moisture stress in an EEO context.

A number of studies have indicated that ecosystems in more arid regions adapt by extracting water at lower rates yet continue to do so at an unchanged rate down to lower critical soil‐moisture thresholds than ecosystems in humid regions. Intuitively, this strategy is consistent with the concept of optimal water use under limited availability. It is expected to be manifested both in evapotranspiration (ET) and GPP due to the dominance of transpiration in ET, and the close coupling between transpiration and GPP. An empirical analysis of the influence of soil moisture stress on ET responses across biomes by Fu et al. (2021) found that critical soil‐moisture thresholds for ET decline vary widely, with arid ecosystems maintaining unchanged ET at lower soil moisture levels than well‐watered ecosystems. Fu et al. (2022) further showed that both the critical soil‐moisture threshold and the maximum evaporative fraction (EF, the fraction of available energy used for ET) under moist conditions are shaped by climatic aridity: more arid ecosystems conserve water at high soil moisture levels, but sustain ET as soil moisture decreases to lower levels. In other words, arid ecosystems optimise water use by adopting strategies that maximise evapotranspiration and photosynthesis under water‐limited conditions. Fu et al. (2024) derived a global map of critical soil‐moisture thresholds, showing that these thresholds vary depending on aridity, soil texture and vegetation cover. Supporting these findings, Bassiouni et al. (2020) identified lower soil‐moisture thresholds for water uptake in arid ecosystems. Theoretical modelling by Manzoni et al. (2014) explains this behaviour as representing optimal coordination of hydraulic traits to maximise long‐term water use, with arid‐adapted plants sustaining water uptake from drier soils. Bassiouni, Manzoni, and Vico (2023) expanded this idea using a theoretical model linking plant hydraulic traits to climate, showing that optimal water‐use strategies result in varied threshold values across climates and demonstrating that these strategies align with an eco‐evolutionary optimal response to water scarcity. These various findings emphasise that vegetation models should incorporate aridity‐related soil‐moisture thresholds and highlight the convergence of data‐driven and theoretical studies in supporting a general hypothesis of optimal water use.

Vegetation and land‐surface models commonly assume that GPP at any location is maximal under well‐watered conditions (Bonan 2019) and account for the effect of low soil moisture on GPP by applying a stress function (β) that reduces GPP—as a function of declining soil moisture—once a critical threshold of soil water availability is reached. However, not all models apply the stress function directly to GPP. Whether a stress function should be applied directly to photosynthesis (as, e.g., in the JULES model: Clark et al. 2011), to stomatal conductance, g_s_ (as in the LPJ model: Sitch et al. 2003) or to the maximum carboxylation rate, *V*_cmax_ (as in the ORCHIDEE model: Krinner et al. 2005) is debated (Rogers et al. 2017; De Kauwe et al. 2013). A more process‐based approach has been implemented in the CLM5 model, using plant hydraulics to predict β as a function of the leaf water potential (Liu et al. 2024). Observational studies show that water stress regulates GPP via both stomatal and non‐stomatal processes (Egea, Verhoef, and Vidale 2011), but open questions remain about their relative importance. In this paper, we focus specifically on how soil moisture influences the LUE of GPP irrespective of specific mechanisms. We derive piecewise linear functions for this relationship on a site‐by‐site basis and show how the parameters of these functions relate to climatic aridity.

The critical soil‐moisture threshold used in the application of soil‐moisture stress functions in current models is either universal or prescribed by vegetation type (e.g., Best et al. 2011; Boussetta et al. 2013; Oleson et al. 2013). However, Fu et al. (2021) using eddy covariance flux tower observations from the ICOS network across Europe, estimated critical soil‐moisture thresholds by analysing EF and soil‐moisture relationships during dry‐down periods and noting how the covariance between VPD and GPP changes sign as soil moisture declines. They showed that the critical soil‐moisture threshold at which EF is reduced varies across biomes and climates. Fu et al. (2022) extended this analysis to a global scale. Their results suggested systematic differences in critical thresholds across ecosystems, with drylands showing adaptations to water scarcity. Comparing grasslands and (dry) savannas, they showed that the EF response of grasslands yields higher annual GPP than if the same ecosystems adopted the EF response of savannas, and *vice versa*. Such findings suggest that models relying on a single threshold underestimate the complexity of plant water use, especially under conditions of water scarcity. These findings are, however, consistent with a shift from isohydric to anisohydric stomatal regulation with increasing climatic aridity (McDowell 2011; Kumagai and Porporato 2012; Konings and Gentine 2017) and with the idea that plant strategies should maximise carbon assimilation over the annual cycle.

Here we compare flux tower–derived estimates of daily GPP, which are inferred from half‐hourly eddy covariance measurements of net ecosystem exchange (NEE) using a variety of assumptions to partition NEE into GPP and respiration components (Pastorello et al. 2020), with the expected ‘well‐watered’ GPP as calculated by the sub‐daily P model (Mengoli et al. 2022) across the full range of aridity represented in the global flux tower network (https://fluxnet.org/). We analysed daily GPP data from 67 eddy‐covariance flux towers representing this range. We fitted breakpoint regressions to account for the impact of soil moisture (θ) on LUE, expressed as the ratio β(θ) of flux‐derived GPP to GPP as predicted by the P model for well‐watered conditions. The observed (remotely sensed) fAPAR, which depends on the leaf area index, was used to drive the sub‐daily P model and is, therefore, already included in the well‐watered GPP simulated by the model. This approach excludes leaf area dynamics, as these are already accounted for in the denominator of the β(θ) ratio (i.e., the well‐watered simulated GPP), in order to focus on LUE.

We then analysed fitted values of both the maximum β(θ) and the critical threshold of θ as non‐linear functions of the climatic aridity index (AI), defined as the ratio of annual potential evapotranspiration (PET) to annual precipitation. These relationships were used to generate a family of β(θ) functions, dependent on AI, which can serve as multipliers of the modelled, well‐watered GPP. The performance of the resulting model was compared with that of the uncorrected sub‐daily P model, with a version of the sub‐daily model that applies the soil‐moisture stress function previously developed by Stocker et al. (2020) for use with the ‘classic’ P model, and with the MODIS remotely sensed GPP product (Running and Zhao 2021). The function relating to the reduction of assimilation due to low soil moisture varies systematically as a function of climatic aridity, rather than being dependent on the type of vegetation. Moreover, GPP reductions under low soil moisture satisfy an optimality criterion: that is, for any given aridity value, there exists a soil moisture level at which the associated GPP response function is maximal; while as aridity increases, this level declines.

## 2 Methods

### 2.1 The P Model

The P model is an LUE model based on EEO theory for the trade‐off between carbon uptake and water loss (Prentice et al. 2014) and the acclimation and/or adaptation of leaf‐level photosynthesis to environmental conditions (Wang et al. 2017). The model is driven by air temperature, VPD, incident photosynthetic photon flux density (PPFD), fAPAR, elevation (through atmospheric pressure) and the ambient partial pressure of carbon dioxide (*c*_a_). The model distinguishes C_3_ and C_4_ photosynthesis but does not require specification of distinct parameter values of any other plant functional types. When driven by satellite‐derived fAPAR, it reproduces the seasonal cycle and interannual variability in GPP at flux sites from a range of natural vegetation types as well as geographic variation in GPP (Balzarolo, Peñuelas, and Veroustraete 2019; Stocker et al. 2020; Wang, Prentice, and Davis 2014) and temporal trends in GPP at flux sites (Cai and Prentice 2020).

The P model was modified by Mengoli et al. (2022) to simulate diurnal cycles, separating the instantaneous responses of GPP (with photosynthetic parameters fixed over the diurnal cycle) from the acclimation responses of those parameters on a time scale of around 2 weeks. This modified model (P‐model subDaily v1.0.0, accessible at Mengoli 2023b) is used here to simulate daily GPP as the daily sum of GPP computed on half‐hourly timesteps. The sub‐daily model can be run in two modes, either by using an exponential‐weighted mean of the acclimating quantities or by using a 15‐day running mean of midday temperature to determine acclimation. The two methods produce virtually identical results (Mengoli et al. 2022). Here, we use a 15‐day running mean of midday temperature to determine acclimation. Mengoli et al. (2022) showed that the P‐model subDaily v1.0.0 accurately reproduces the diurnal cycle of GPP in well‐watered sites but overestimates GPP in drylands because it does not include any soil‐moisture limitation on GPP.

The FULL configuration of the current standard P model Pv1.0 (Stocker et al. 2020) includes an empirical water stress function (also based on eddy‐covariance flux data) that approaches 1 at a threshold value of θ (θ*), where θ is plant‐available water expressed as a fraction of soil water‐holding capacity, and θ* is set to 0.6. The function declines more steeply with decreasing θ in drier climates, with climatic moisture quantified by an estimate of the ratio (α) of actual evapotranspiration (AET) to potential evapotranspiration (PET). This function is used in Pv1.0 (FULL) as a multiplier of the modelled, well‐watered GPP, in a similar way to the function proposed here (accessible at Mengoli 2023a) but has not previously been applied in the sub‐daily model.

### 2.2 Flux Tower Data

GPP and meteorological data at 67 flux tower sites (Table S1) were obtained from the FLUXNET2015 dataset (Pastorello et al. 2020). We used GPP based on the daytime partitioning method (Lasslop et al. 2010; Pastorello et al. 2020). FLUXNET2015 provides the meteorological variables required to run the P model, including air temperature, VPD and PPFD on a half‐hourly timestep. However, it does not provide fAPAR. We obtained fAPAR at each site from the dataset produced by Stocker et al. (2020) from the MODIS MCD15A3H Collection 6 dataset (Myneni, Knyazikhin, and Park 2015), accessible at Stocker (2020). The original dataset has a spatial resolution of 500 m and a temporal resolution of 4 days. Stocker et al. (2020) filtered these data to remove points where clouds were present and derived daily data by linear interpolation. We used a subset of the sites from Stocker et al. (2020), chosen to cover the full range of aridity with no major gaps. We initially selected all sites available in the FLUXNET2015 dataset that, according to the 20‐year climatological aridity index (see paragraph below for its computation), were classified as arid/dry sites—nine in total. Then, we included 22 sites classified as semi‐arid and 36 sites classified as humid. To balance the contributions from arid, semi‐arid and humid categories, we intentionally did not include all available sites in FLUXNET2015, as the excluded sites were all classified as humid. Sites were selected based on the following criteria: geographic distribution to ensure sites were spread out globally across different climates and representativeness of the full range of vegetation types in the dataset. Sites were also selected based on a minimum record length of 2 years, with quality‐control flags indicating ‘good’ observations for at least 80% of the half‐hourly records (Table S1). For arid sites, however, the 2‐year minimum record requirement was not applied due to the limited number of such sites, which would have further reduced the number of arid sites available for analysis. Meteorological and MODIS data were not available for some sites/years, so analyses and simulations were based on different years across sites (Table S1). Only the half‐hourly records flagged as ‘good’ were used.

### 2.3 Calculation of the GPP Reduction Factor

We calculated the ratio β(θ) between flux‐derived and P‐model subDaily v1.0.0 modelled, well‐watered GPP for each site and day. Our approach differs from that of Stocker et al. (2020) in three key respects. First, our fitted stress function is allowed to take values < 1 under well‐watered conditions. We thus allow for the possibility that ecosystems adapted to arid climates use water more conservatively even when soil moisture is abundant. Second, in order to ensure consistency of the soil moisture calculation across sites, we calculate daily soil moisture using the Simple Process‐led Algorithms for Simulating Habitats (SPLASH) model (version 1: Davis et al. 2017) with simulated soil moisture converted to relative soil water content (θ) by dividing by the generic bucket size in SPLASH (150 mm). Third, we use the aridity index AI (the ratio of PET to annual precipitation) rather than the factor α used by Stocker et al. (2020) as a climatological index, because of its wider use in the literature, and because its calculation is independent of the SPLASH model's estimation of ΑΕΤ.

### 2.4 Breakpoint Regression Analysis

We used breakpoint regression (Toms and Lesperance 2003) to evaluate the relationship between the β(θ) ratio and soil water content, which identifies and estimates the maximum level of the β(θ) ratio under well‐watered conditions, and the critical threshold below which the ratio declines linearly towards the wilting point, at each site. This model was selected based on its ability to capture key transitions in water stress, as demonstrated in a previous study (Fang and Gentine 2024) where a piecewise linear relationship was found to consistently represent water stress limitations across diverse ecosystems. Before this analysis, we removed values of flux‐derived GPP below the 5th percentile (which gave highly variable β(θ) ratios) and observations with greater than the 99th percentile of θ, which would otherwise have dominated the regression at many well‐watered sites. Preliminary analyses showed that the intercept was generally close to zero and that imposing the constraint β(0) = 0 had little effect at the great majority of sites (Figure S1). We therefore imposed this constraint resulting in a regression model with just two parameters, the maximum level of β(θ) (*y*) and the critical threshold of θ (ψ):

(1) $$\beta \left(\theta \right)=\min\;\left[y,\left(y/\psi \right)\times \theta \right]$$

where β(θ) is equal to its maximum level (*y*) when θ ≥ ψ while it is equal to the ratio between its maximum level and the critical threshold (*y*/ψ) when θ < ψ.

The non‐parametric Kruskal–Wallis test was used to determine whether there were significant differences in fitted parameter values among aridity classes. We used *p* < 0.05 as the criterion to identify significant differences between classes.

### 2.5 Calculation of the Aridity Index

The length of the meteorological records in FLUXNET2015 is too short to calculate a climatological index at most sites. We, therefore, derived AI using climate data for a 20‐year period (2001–2020) from the CRU TS 4.06 gridded climate dataset (Harris et al. 2020). We obtained precipitation data directly from the CRU dataset and calculated PET using temperature, precipitation and cloud cover from this dataset as inputs to SPLASH version 1 (Davis et al. 2017). Of the 67 selected sites, nine were classed as arid (AI > 5), 22 as semi‐arid (2 < AI < 5) and 36 as humid (AI < 2) (Tables 1 and S1). We removed two sites classified as arid (AU‐Lox, AI = 6.32, and US‐Wkg, AI = 6.34) and one classified as semi‐arid (AU‐RDF, AI = 2.16), either because they were irrigated crops (AU‐Lox, AU‐RDF) or because the presence of extensive wetlands indicates that they were groundwater‐fed (US‐Wkg). The derivation of the stress function was thus eventually based on the analysis of 64 sites.

**TABLE 1:** Statistics of P model performance (root mean squared error, RMSE and percent bias, PBIAS) using the new soil‐moisture stress function (new), with its aridity‐based parameters, and the stress function used by Stocker et al. (2020) but applied in the sub‐daily model used here, compared to P model performance with no soil moisture correction (ww). The sites are grouped by aridity index (AI) classes (see also Supplementary Table 1).

Site ID	AI	ARIDITY	RMSE(ww)	RMSE(new)	RMSE(v1.0)	PBIAS (ww)	PBIAS (new)	PBIAS (v1.0)
AU‐TTE	7.17	arid	2.07	0.51	0.94	2938.9	658.4	1299.3
AU‐ASM	6.97	arid	2.47	0.96	1.02	277.1	−3.3	99.5
AU‐Cpr	6.36	arid	2.83	0.77	0.87	187.6	−27.4	37.7
US‐Wkg	6.34	not used	3.93	0.9	1.86	349.6	15.1	145.3
AU‐Lox	6.32	not used	2.15	7.03	5.79	2	−76.1	−58.9
US‐Whs	5.89	arid	3.4	0.93	1.68	571.7	74.3	266.8
AU‐GWW	5.75	arid	2.57	0.53	1.1	197.4	−18.7	70.1
US‐SRG	5.08	arid	4.01	1.46	2.25	289.4	7.1	135.9
US‐SRM	5.02	arid	2.82	1.04	1.45	246.5	−5.1	106.6
US‐Cop	3.99	semi‐arid	1.89	0.46	1.05	577.9	85.8	300.3
AU‐Ync	3.96	semi‐arid	2.75	0.67	1.7	428.9	53.5	240.1
ES‐Ln2	3.84	semi‐arid	3.92	0.77	1.71	5359.2	1096.2	2468.2
AU‐Stp	3.71	semi‐arid	2.62	1.33	1.44	162.5	−16	79.3
AU‐Emr	3.08	semi‐arid	4.39	1.03	2.87	320.5	50.3	198.8
AU‐Gin	2.93	semi‐arid	3.22	1.61	1.71	89.9	−41.2	32.8
AR‐SLu	2.89	semi‐arid	2.07	5	2.13	16.9	−56.3	−13.8
ES‐LgS	2.88	semi‐arid	3.33	0.78	1.69	197.4	−9.2	99.3
CN‐Du2	2.7	semi‐arid	4.53	1.47	3.02	421.4	87.9	256.7
ZA‐Kru	2.69	semi‐arid	2.14	3.3	1.82	19.5	−55.5	−7.5
US‐AR2	2.61	semi‐arid	3.88	1.39	2.59	318	61.1	205.5
US‐AR1	2.49	semi‐arid	3.1	1.5	2.15	156.2	2.9	89.8
AU‐Whr	2.39	semi‐arid	3.13	1.41	1.63	79.6	−35.4	36.2
CN‐HaM	2.34	semi‐arid	1.63	1.68	1.02	48.6	−41.1	18
AU‐Dry	2.32	semi‐arid	3.31	1.85	1.63	85.6	−36.6	38.3
IT‐Noe	2.26	semi‐arid	4.04	1.61	1.86	99	−38.4	42.8
US‐Ton	2.23	semi‐arid	4.39	1.4	3.05	140.9	−19.4	85.8
US‐Var	2.22	semi‐arid	5.6	1.27	4.01	313	40.3	219.3
ZM‐Mon	2.18	semi‐arid	3.11	3.2	1.88	38.5	−50.3	8.9
AU‐RDF	2.16	not used	4.34	2.3	3.46	194.7	11	140
US‐ARb	2.04	semi‐arid	4.02	2.91	3.05	100.7	−13.8	63.1
US‐ARc	2.04	semi‐arid	3.46	2.54	2.43	80.4	−22.9	46.1
AU‐DaS	1.81	humid	2.3	2.9	1.56	29	−50.8	5.8
AU‐Rig	1.81	humid	3.91	1.81	3.45	106.6	−7.8	86.9
AU‐DaP	1.8	humid	3.76	3.21	2.66	70.4	−32.8	43.7
AU‐Wom	1.75	humid	5.63	2.26	4.25	65.2	−25.7	47
IT‐Cp2	1.73	humid	6.05	2.49	4.1	76.1	−29.4	47.4
AU‐Wac	1.69	humid	3.79	2.54	2.54	41.8	−39.8	20.9
FR‐Pue	1.57	humid	5.22	1.56	3.6	116.7	−14.8	81.7
AU‐Ade	1.55	humid	2.3	3.5	1.88	8.4	−52.2	−3.8
AU‐How	1.46	humid	2.83	3.23	2.01	23.3	−53.2	2
CA‐SF3	1.41	humid	4.38	1.12	3.61	161.7	30.5	134.7
FR‐Fon	1.39	humid	3.04	3.39	2.59	42.1	−34	26
IT‐Col	1.35	humid	4.95	3.32	3.59	79.3	−24.3	53.5
AU‐Tum	1.34	humid	4.51	3.78	3.76	33.2	−32.9	23.4
IT‐SRo	1.34	humid	4.34	2.75	2.9	53.1	−34.6	34.6
US‐KS2	1.21	humid	13.08	5.23	12.65	233.6	84.3	226
CA‐Man	1.19	humid	5.38	2.06	4.94	160.4	40.7	144.5
CA‐NS4	1.19	humid	4.09	1.48	3.82	150.8	39.4	140.5
DE‐Gri	1.18	humid	2.32	2.87	2.07	17.1	−35.3	12.1
IT‐MBo	1.18	humid	4.51	2.13	4.09	69.1	−2.7	62.4
RU‐Ha1	1.11	humid	1.75	1.05	1.58	46.4	−17.2	39.8
FR‐LBr	1.1	humid	3.27	2.18	2.56	40.4	−31	29.9
US‐Wi6	1.08	humid	5.5	2.18	5.46	177.2	66	176.2
AR‐Vir	1.02	humid	4.24	2.9	3.87	35.5	−20.7	31.2
US‐PFa	1.02	humid	4.33	1.91	4.26	146.3	50.7	144.2
US‐Syv	1.01	humid	4.88	2	4.84	87.6	15.6	86.6
RU‐Fyo	0.97	humid	2.92	2.14	2.79	36.3	−19.7	32.9
BE‐Bra	0.91	humid	3.01	1.32	3	45.8	−5.8	45.7
FI‐Hyy	0.87	humid	2.96	1.97	2.86	50.1	−10.6	47.3
NL‐Hor	0.84	humid	3.31	1.73	3.14	65.5	−4.5	62.4
CH‐Oe1	0.8	humid	3.67	3.94	3.67	2.8	−31.5	2.6
BR‐Sa3	0.78	humid	11.1	5.04	11.03	105.3	34.1	104.6
CZ‐BK2	0.78	humid	5.74	3.16	5.73	89.3	31.1	89.1
DE‐RuR	0.78	humid	6.42	2.96	6.4	101.1	36.1	100.8
BE‐Vie	0.73	humid	2.54	2.33	2.54	14.3	−22.5	14.3
CH‐Fru	0.71	humid	7.17	3.85	7.17	97.8	43.5	97.8
IT‐Tor	0.63	humid	3.83	2.14	3.83	80.3	37.2	80.3

### 2.6 Dependencies of Parameters on Aridity

The breakpoint regression yielded values of two parameters (*y*, ψ) for each of the 64 sites. We fitted relationships for each parameter as functions of site AI using non‐linear regression. Both parameters were fitted with a power function:

(2) $$\text{parameter}=\min\;\left[a⸱{\mathrm{AI}}^{b},1\right]$$

where *b* is expected to be negative. This function is bounded above in order to avoid potential values > 1 in extremely wet sites, although none were present in the dataset.

### 2.7 Application

Equations (1) and (2) determine a unique β(θ) function for each value of AI. This function was applied as a multiplier of modelled GPP:

(3) $${\mathrm{GPP}}_{\mathrm{new}}={\mathrm{GPP}}_{\mathrm{ww}}\times \beta \left(\theta \right)$$

where GPP_new_ is the revised, soil‐moisture corrected GPP, GPP_ww_ is the GPP simulated by the P‐model subDaily v1.0.0 without soil‐moisture correction and β(θ) is given by Equation (1) with parameter values derived from Equation (2) as a function of site AI. We compared the predictions of GPP obtained using this new soil‐moisture stress function to GPP simulated by the P‐model subDaily v1.0.0 with (a) no soil‐moisture stress and (b) the soil‐moisture stress function used in Pv1.0 at all of the flux tower sites, with meteorological data provided for the site in the FLUXNET2015 dataset and fAPAR data from Stocker et al. (2020). We also compared these predictions with the current, improved version of the widely used MODIS GPP product (MOD17A2HGF v0.61: Running and Zhao 2021; https://doi.org/10.5067/MODIS/MOD17A2HGF.061). The goodness of fit between each of the modelled estimates of GPP and the flux‐derived GPP at each site was quantified by the root mean squared error (RMSE).

## 3 Results

**FIGURE 1:**
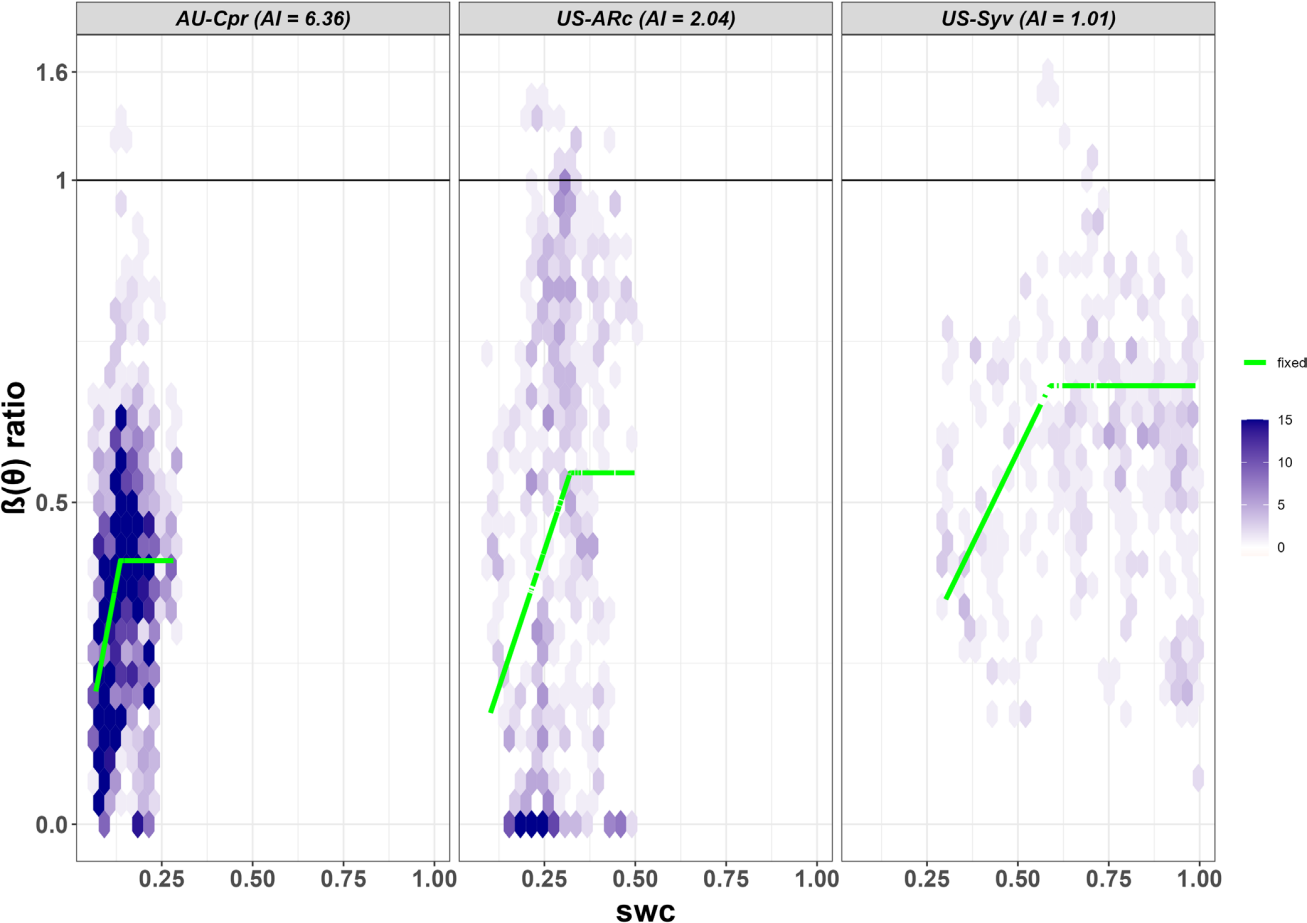
Relationship between soil moisture stress and the β(θ) ratio. Examples of the fitted maximum β(θ) ratio (the ratio of actual flux‐derived to modelled well‐watered gross primary production) and its response to relative soil moisture below the critical threshold (green line) for three sites representing the range of climatological aridity levels using site‐specific parameters. The β(θ) ratio and relative soil water content are both unitless. Note that the scale above 1 has been compressed for visualisation purposes. Plots for all the sites used in the analysis are given in Figure S2.

The response of LUE to water stress could be described by Equation (1) using site‐specific parameters (Figures 1 and S2). To assess the fit of the breakpoint regression model across sites, we computed R‐squared values, included in Figure S2. As expected, the R‐squared values are variable, reflecting site‐specific environmental variability and inherent uncertainties. Despite this variability, the selection of the breakpoint regression model is supported by previous research (Fang and Gentine 2024), which demonstrated that a piecewise linear relationship is appropriate for describing water stress limitation across diverse ecosystems. These findings provide a solid theoretical foundation for applying the model to estimate GPP under varying moisture conditions.

**FIGURE 2:**
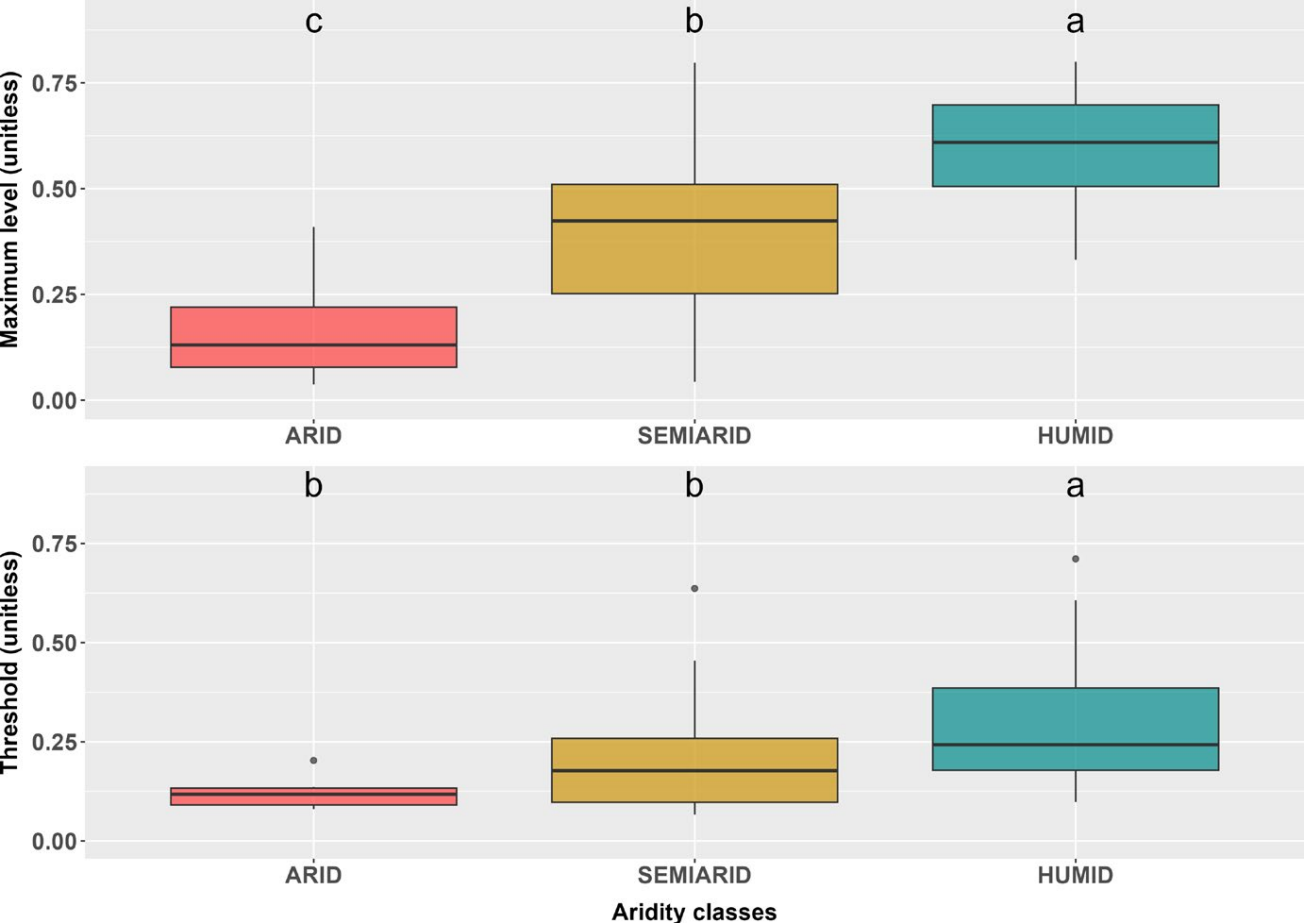
Comparison of the maximum β(θ) ratio and the critical threshold value of soil moisture. Box‐plot comparison of the fitted maximum β(θ) ratio (the ratio of actual flux‐derived to modelled well‐watered gross primary production) (above) and the critical threshold value of soil moisture (below) under arid, semi‐arid and humid conditions, using site‐specific parameters. Arid sites have AI > 5, semi‐arid sites have AI between 2 and 5, and humid sites have AI < 2. The black line is the median, the box is the interquartile range and the whiskers show the range, with outliers shown as asterisks. Letters indicate whether the median values are significantly different based on the Kruskal–Wallis test, *p* < 0.05. Classes that are significantly different from one another are indicated by different letters.

**FIGURE 3:**
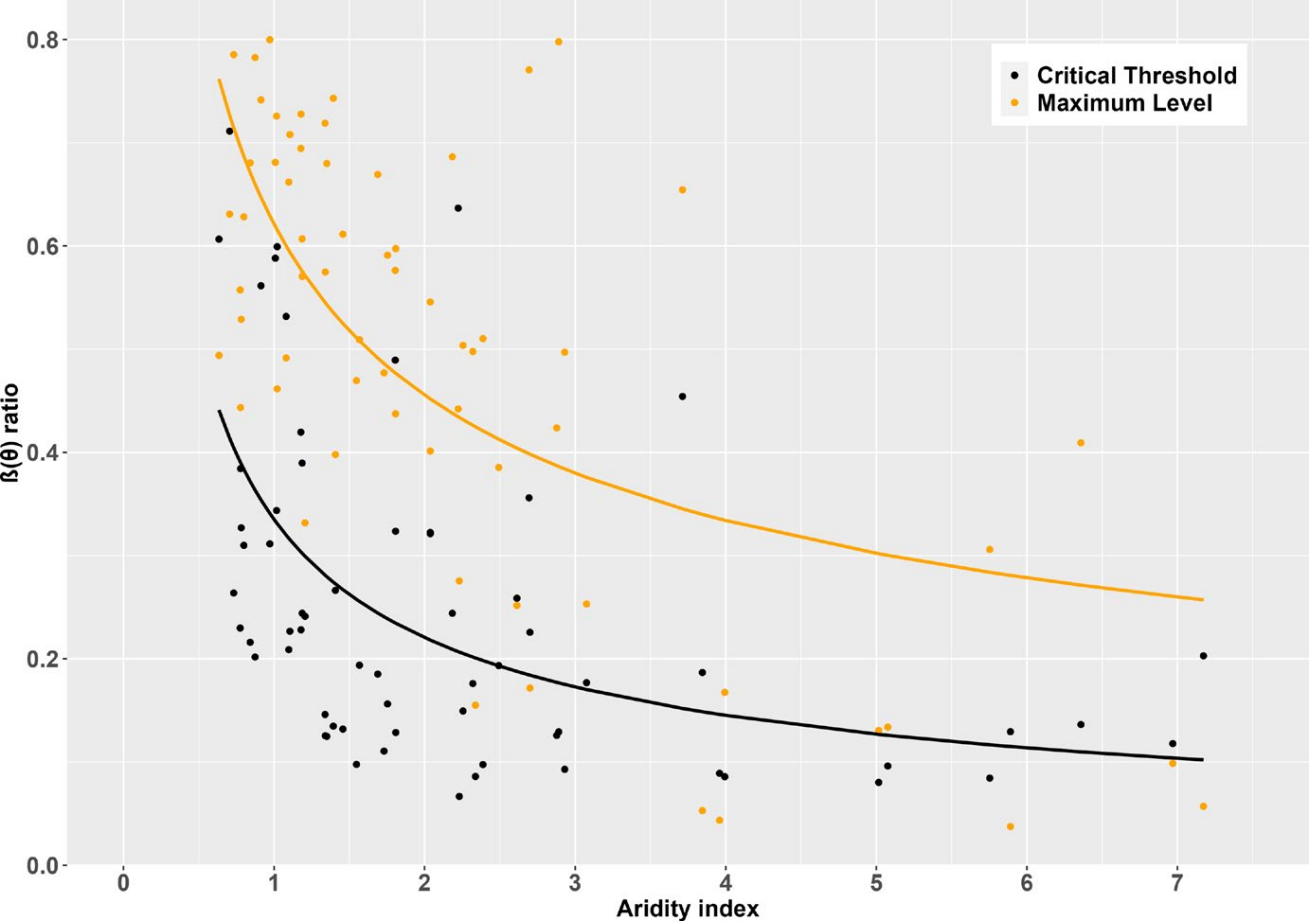
Maximum β(θ) ratio and the critical soil‐moisture threshold under different aridity levels. Values of the fitted maximum β(θ) ratio (the ratio of actual flux‐derived to modelled well‐watered gross primary production) using site‐specific parameters and the critical threshold value of soil moisture against the climatic aridity index (AI), showing non‐linear regressions of both parameters against AI.

Figures 2 and 3 depict site‐specific parametrisation results, showing the dependence of GPP reduction thresholds and maximum assimilation values on aridity conditions. Both the maximum assimilation level and the critical threshold at which soil moisture stress starts to impact LUE were found to vary systematically with aridity. The maximum assimilation level under well‐watered conditions becomes progressively lower from humid through semi‐arid to arid sites (Figure 2).

The difference between humid, semi‐arid and arid sites is significant. The critical threshold is also reduced, such that water stress sets in at higher soil moisture in humid sites than in semi‐arid or arid sites (Figure 2). This difference is also significant. Moreover, the slope of the stress function below the critical threshold becomes progressively steeper with increasing aridity. Thus, plants growing in more arid environments have a lower maximum LUE overall but sustain this level under drier soil conditions (Figure 3). These relationships were also evident when the intercept was not constrained to zero (Figure S3).

**FIGURE 4:**
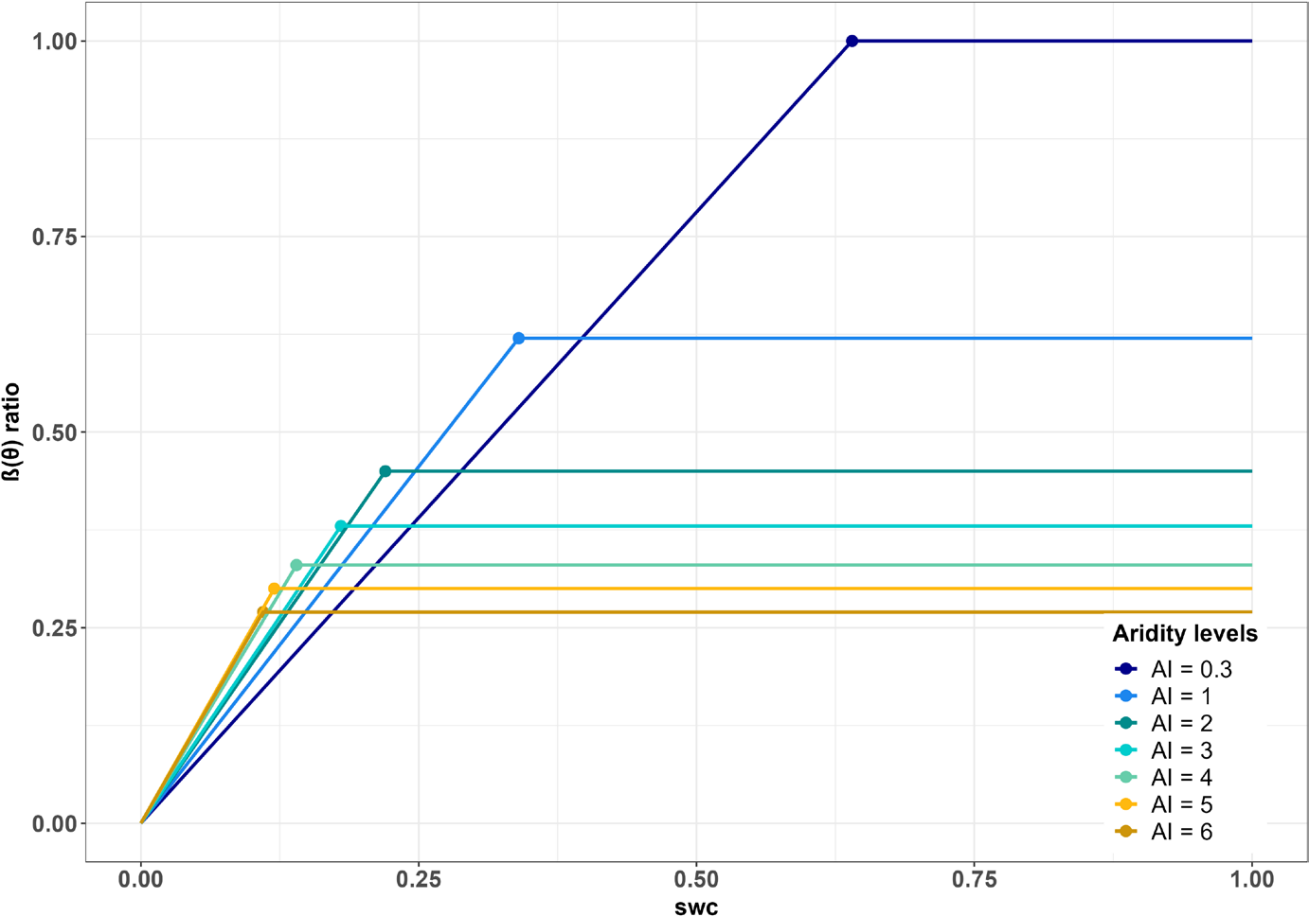
Predicted β(θ) ratio functions for different aridity levels. Predicted β(θ) ratio (the ratio of actual flux‐derived to modelled well‐watered gross primary production) functions based on the regressions shown in Figure 3, for different levels of the aridity index (AI).

Both model parameters showed non‐linear relationships with AI that could be fitted using Equation (2) (Figure 4). Although there were some outliers, these do not seem to be related to either vegetation type (Figure S4) or the seasonal concentration of precipitation (Figure S5). The derived equations for the maximum β(θ) level (*y*) and the critical threshold of θ (ψ) are as follows:

(4) $$y=\min\;\left[0.62\;{\mathrm{AI}}^{–0.45},1\right]$$

and

(5) $$\psi =\min\;\left[0.34\;{\mathrm{AI}}^{–0.60},1\right]$$

**FIGURE 5:**
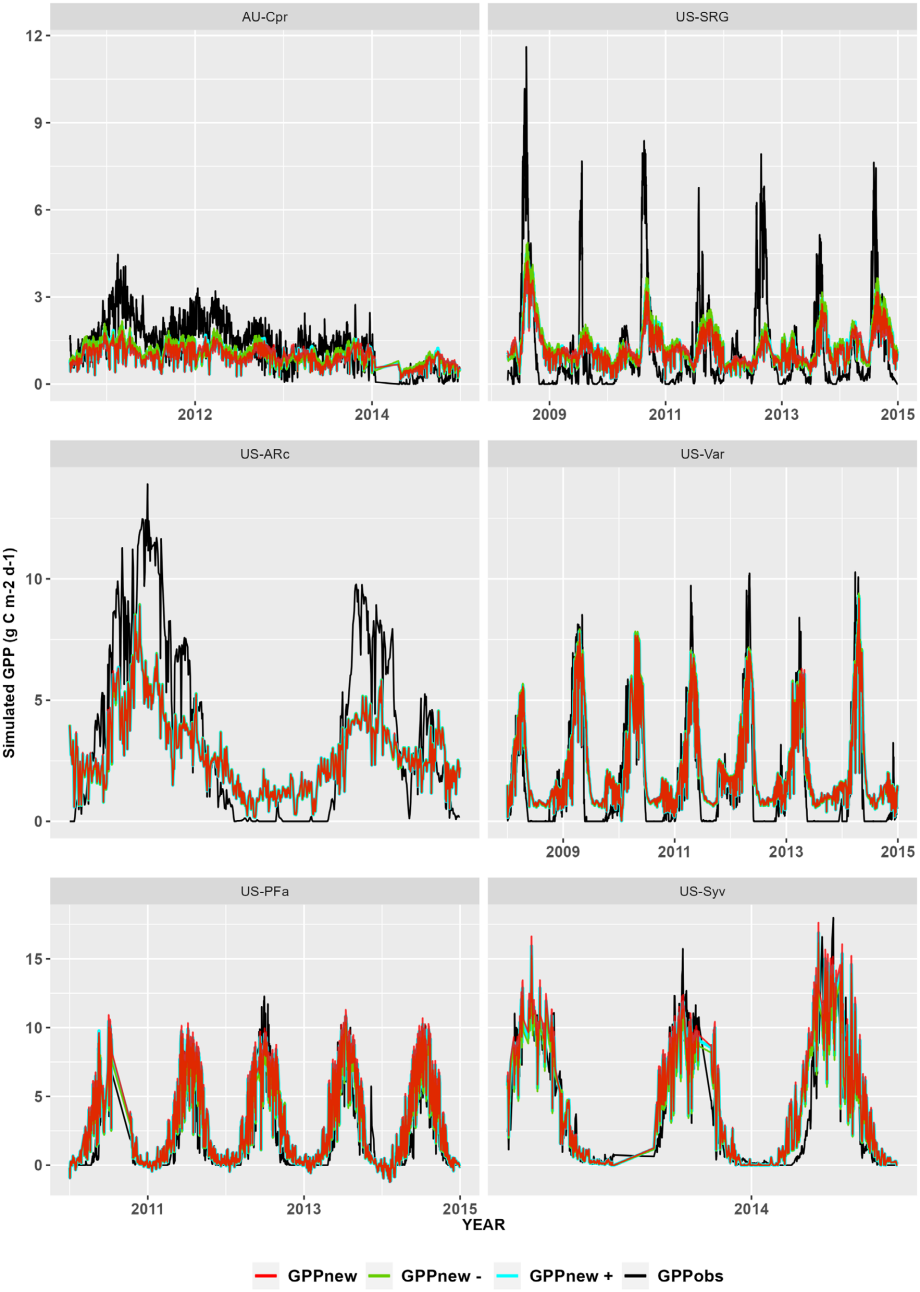
Sensitivity of the model to parameter uncertainty. The plot shows gross primary production (GPP) using the new soil‐moisture stress function (GPPnew) at six sites representing the range of climatological aridity compared to the simulated GPP resulting from adding the upper (GPPnew +) and lower (GPPnew −) standard error to the canonical fitted parameters in Equations 4 and 5. The flux‐derived values (GPPobs) are also shown. Note that the scale varies between the rows.

We performed a sensitivity test to assess the impact of uncertainty in the estimated parameters on GPP by substituting the upper and lower values of the standard errors on the fitted parameters in Equations 4 and 5. This test showed that these uncertainties had little impact on β(θ) and did not change the simulated GPP (Figure 5).

**FIGURE 6:**
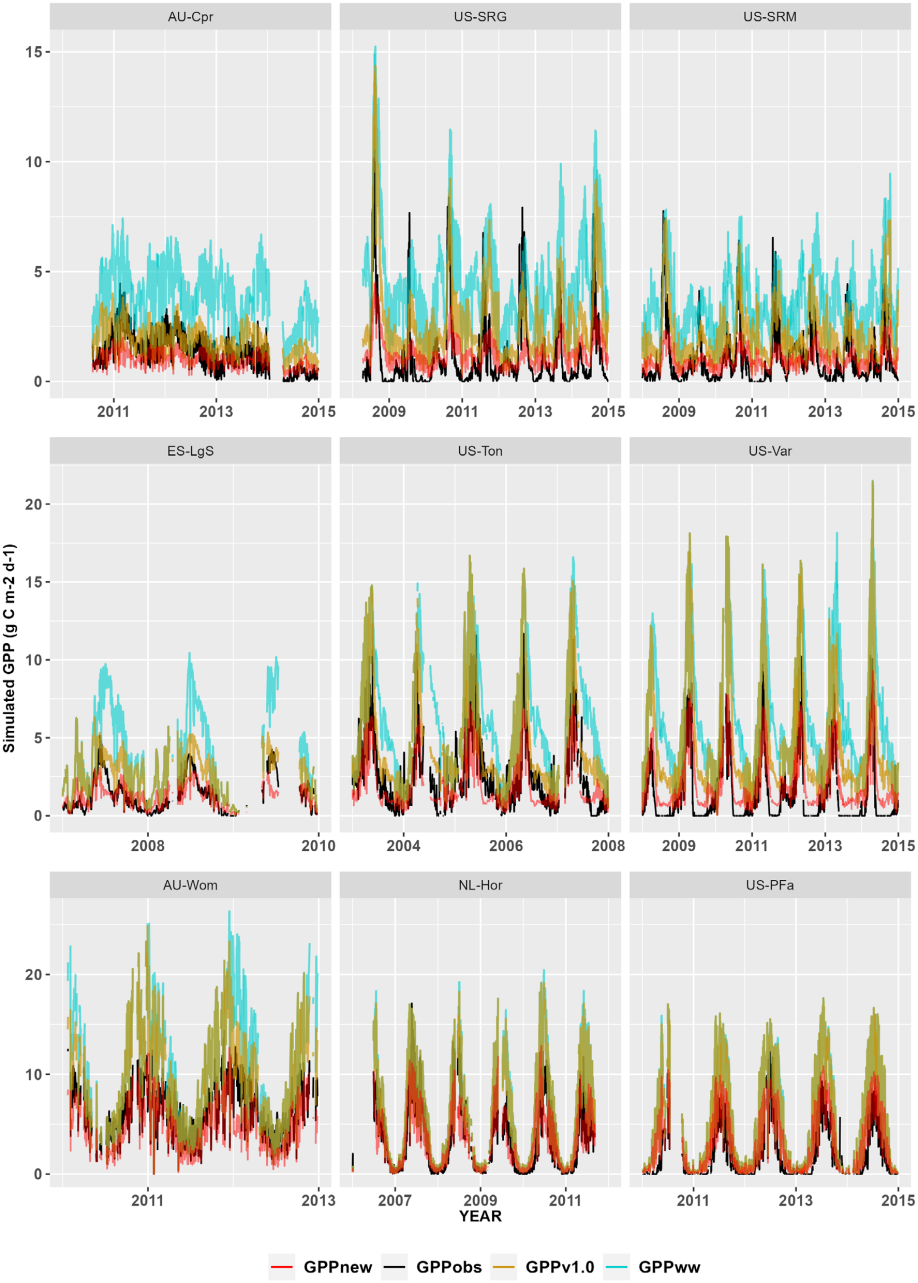
Impact of new soil‐moisture stress function on simulated gross primary production. Examples of how the new soil‐moisture stress function, with its aridity‐based parameters, modifies simulated gross primary production (GPP_new_) at nine sites representing the range of climatological aridity compared to how the original stress function, when applied in the sub‐daily model, affects simulated GPP (GPPv1.0). The new model is compared to the simulated level of GPP under well‐watered conditions (GPP_ww_), and to flux‐derived values (GPP_obs_). Note that the scale varies between the rows. Plots for all the flux tower sites are given in Figures S6–S11.

Implementation of the new empirical soil‐moisture stress function, using aridity‐based parameters (i.e., the same set of parameters, in Equations 4 and 5, applied to all sites), produced a substantial improvement in model performance compared to simulations with no soil‐moisture stress function (Figures 6 and S6–S8). At sites classified as arid (AI > 5), simulations that did not account for soil–water stress produced an overestimation of maximum GPP between 2 and 8 gC m^−2^ d^−1^ (The only exception to this was AU‐Lox where the P model predictions that did not account for soil‐water stress accurately matched the observed magnitude of GPP; see Figure S4. This site is an irrigated orchard). The overestimation of peak GPP at sites classified as semi‐arid (AI between 2 and 5) was of a similar magnitude (2 to 10 gC m^−2^ d^−1^). Even at sites classified as humid (AI < 2), there was an improvement in performance at most sites (Figures 6 and S8).

To further illustrate the accuracy of the model, we present a heatmap scatter plot comparing simulated and observed daily GPP at the same selected sites displayed in Figure 6 (Figure S9). This visualisation provides a visual assessment of model accuracy relative to the 1:1 line, with denser concentrations of data points appearing in red. The model shows the strongest agreement for lower GPP values, where the highest data density is concentrated, while discrepancies emerge at higher GPP values. R^2^ values for these sites confirm this pattern, with poor values in the three arid sites—AU‐Crp, US‐SRM and US‐SRG—and in the semi‐arid site (ES‐LgS), but higher values for the other five sites—US‐Var (0.76), US‐Ton (0.70), AU‐Wom (0.67), NL‐Hor (0.75) and US‐PFa (0.75), demonstrating good predictive skills in humid and semi‐arid regions.

The improved performance compared to the version of the P model with no soil‐moisture stress function is reflected in the RMSE values (Table 1). The RMSE for arid sites ranged from 0.51 to 1.46 gC m^−2^ d^−1^ compared to 2.07 to 4.01 gC m^−2^ d^−1^ when no stress function was applied. All of the arid sites showed a reduction in RMSE, with an average reduction in RMSE of 69.3%. The RMSE for semi‐arid sites ranged from 0.46 to 5.0 gC m^−2^ d^−1^ compared to 1.63 to 5.6 gC m^−2^ d^−1^ when no stress function was applied. All but four of the 21 semi‐arid sites showed a reduction in RMSE, with an average reduction in RMSE of 47.3%. The RMSE for humid sites ranged from 1.05 to 5.23 gC m^−2^ d^−1^ compared to 1.75 to 13.08 gC m^−2^ d^−1^ when no stress function was applied. All but five of the 36 humid sites showed a reduction in RMSE, with an average reduction of 42.1%.

The new soil‐moisture stress function also performed substantially better than the stress function used in Pv1.0, reducing the overestimation of peak GPP across arid, semi‐arid and humid sites (Figures 6 and S10–S12). The RMSE for arid sites ranged from 0.51 to 1.46 gC m^−2^ d^−1^ compared to 0.87 to 2.25 gC m^−2^ d^−1^ when the Pv1.0 moisture‐stress function was applied. All of these sites showed reduced RMSE. The RMSE for semi‐arid sites ranged from 0.46 to 5.0 gC m^−2^ d^−1^ compared to 1.02 to 4.01 gC m^−2^ d^−1^ when the Pv1.0 moisture‐stress function was applied. All but six of these 22 sites showed reduced RMSE. The RMSE for humid sites ranged from 1.05 to 5.23 gC m^−2^ d^−1^ compared to 1.56 to 12.65 gC m^−2^ d^−1^ when the Pv1.0 moisture‐stress function was applied. All but eight of these 36 sites showed reduced RMSE.

**FIGURE 7:**
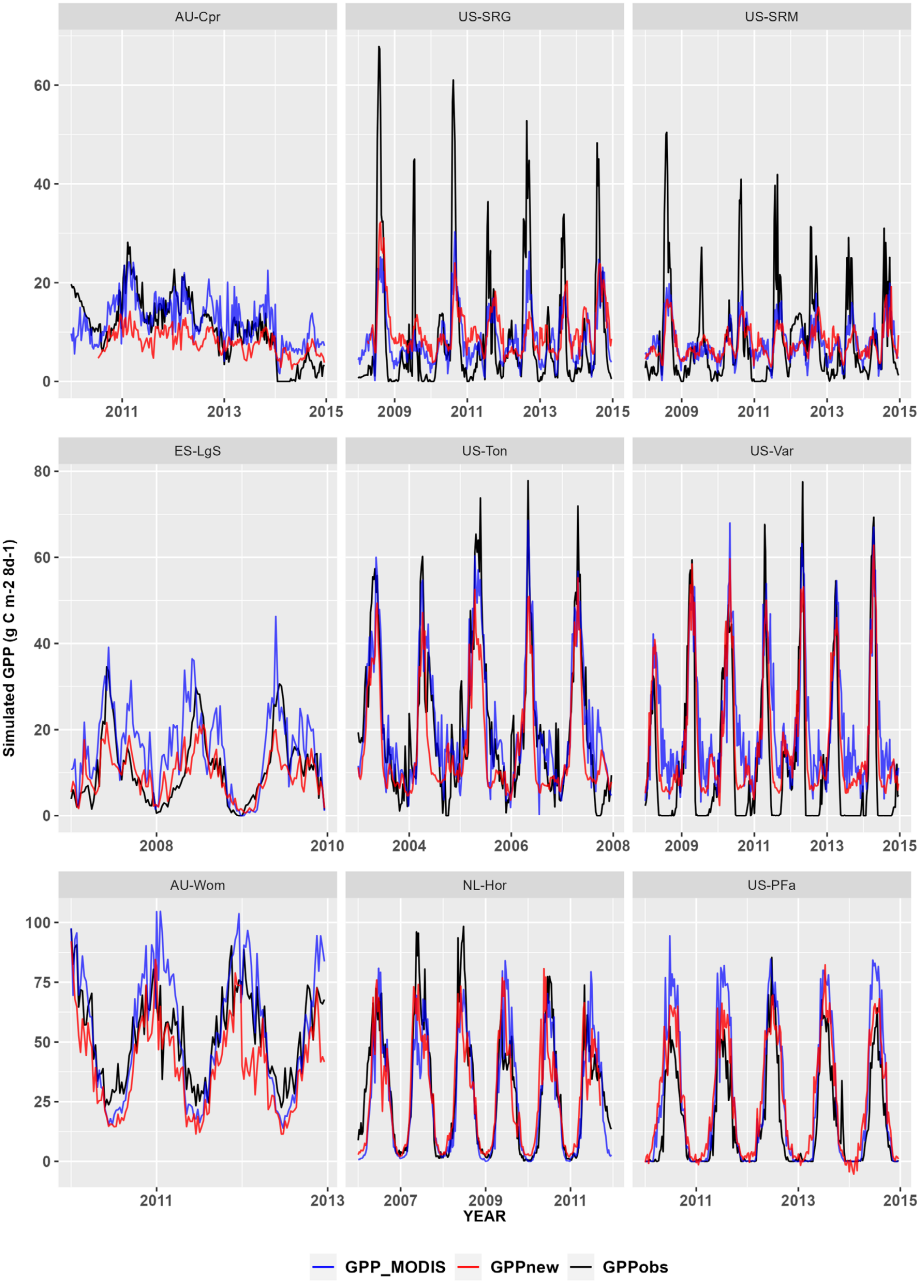
Comparison of simulated and observed gross primary production under different aridity levels. Comparison of simulated gross primary production, including the new soil‐moisture stress function (GPPnew), with its aridity‐based parameters, and the gross primary production simulated by MOD17A2HGF v0.61 (GPP_MODIS_) against flux‐derived values (GPPobs) at nine sites representing the range of climatological aridity. Note that the scale varies between the rows. Plots for all the flux tower sites are given in Figures S12–S14.

Comparison of the new soil‐moisture stress function with MODIS GPP shows a similar level of performance (Figure 7; Supplementary Figures 13–15). The average RMSE for the P model and MODIS at arid sites is 6.67 and 5.80 gC m^−2^ 8‐d^−1^, respectively, and the range of RMSE values (Table 2) is comparable (3.51–11.08 gC m^−2^ 8‐d^−1^ for the P model; 3.50–9.83 gC m^−2^ 8‐d^−1^ for MODIS GPP). The average RMSE at semi‐arid sites is 13.98 and 14.66 gC m^−2^ 8‐d^−1^ for the P model and MODIS, respectively, with ranges 3.84–39.41 gC m^−2^ 8‐d^−1^ (P model) and 4.62–51.60 gC m^−2^ d^−1^ (MODIS GPP). The average RMSE at humid sites is 19.61 and 16.80 gC m^−2^ 8‐d^−1^ for the P model and MODIS, respectively, and again the ranges are comparable (P model: 6.17 to 40.95 gC m^−2^ 8‐d^−1^; MODIS 6.40 to 30.06 gC m^−2^ 8‐d^−1^).

**TABLE 2:** Statistics of P model performance (root mean squared error, RMSE and percent bias, PBIAS) using the new soil‐moisture stress function (new), with its aridity‐based parameters, compared to MOD17A2HGF v0.61 performance (MODIS). The sites are grouped by aridity index (AI) classes (see also Table S1).

Site ID	AI	ARIDITY	RMSE(new)	RMSE(MODIS)	PBIAS (new)	PBIAS (MODIS)
AU‐TTE	7.17	arid	4.01	4.27	702.7	692.2
AU‐ASM	6.97	arid	7.51	5.96	−2.2	20.7
AU‐Cpr	6.36	arid	5.51	4.45	−24.7	18.3
US‐Wkg	6.34	not used	6.96	6.78	14.2	−19.4
AU‐Lox	6.32	not used	52.43	41.96	−75.5	−57
US‐Whs	5.89	arid	7.19	5.49	74.6	32.7
AU‐GWW	5.75	arid	3.51	3.45	−18.1	7.4
US‐SRG	5.08	arid	11.08	9.83	7.4	−7.1
US‐SRM	5.02	arid	7.86	7.13	−5.4	−4.6
US‐Cop	3.99	semi‐arid	3.84	4.62	112.2	145.2
AU‐Ync	3.96	semi‐arid	7.99	9.91	156.1	241.9
ES‐Ln2	3.84	semi‐arid	6.86	9.92	1080.4	1594.5
AU‐Stp	3.71	semi‐arid	10.11	7.62	−15.4	19.8
AU‐Emr	3.08	semi‐arid	7.75	11.1	47.6	84.8
AU‐Gin	2.93	semi‐arid	12.56	8.6	−40.8	18.1
AR‐SLu	2.89	semi‐arid	39.41	51.59	−56.3	−74.2
ES‐LgS	2.88	semi‐arid	5.49	8.27	−10.7	47.7
CN‐Du2	2.7	semi‐arid	11.15	9.28	89.8	63.8
ZA‐Kru	2.69	semi‐arid	23.02	15.19	−51.3	12
US‐AR2	2.61	semi‐arid	10.04	9.05	61.2	15.2
US‐AR1	2.49	semiarid	15.48	15.85	−17.4	−29.1
AU‐Whr	2.39	semi‐arid	10.64	6.86	−35.3	−9.2
CN‐HaM	2.34	semi‐arid	12.64	14.71	−40.8	−56.8
AU‐Dry	2.32	semiarid	15.49	12.06	−40.5	−17.6
IT‐Noe	2.26	semi‐arid	12.55	19.84	−39.7	49.7
US‐Ton	2.23	semi‐arid	10.02	8.71	−21	5.1
US‐Var	2.22	semi‐arid	9.52	13.97	39.2	64.4
ZM‐Mon	2.18	semi‐arid	24.53	17.68	−50.2	−14.7
AU‐RDF	2.16	not used	17.93	23.67	−1.6	28.7
US‐ARb	2.04	semi‐arid	25.27	29.53	−21.4	−34
US‐ARc	2.04	semi‐arid	19.62	23.62	−22.9	−32.8
AU‐DaS	1.81	humid	21.7	22.01	−48.1	−31.3
AU‐Rig	1.81	humid	13.51	14.97	−6.2	−2.3
AU‐DaP	1.8	humid	24.87	23.36	−32.1	−6.4
AU‐Wom	1.75	humid	15.75	14.04	−23.7	4.7
IT‐Cp2	1.73	humid	22.62	14.21	−33.5	−2.5
AU‐Wac	1.69	humid	19.49	20.18	−37.3	19
FR‐Pue	1.57	humid	11.57	10.92	−14.6	8.2
AU‐Ade	1.55	humid	26.83	24.76	−52.8	−43
AU‐How	1.46	humid	24.62	17.08	−51.2	−22
CA‐SF3	1.41	humid	6.17	12.08	8.2	23.8
FR‐Fon	1.39	humid	26.27	12.05	−34.2	3.6
IT‐Col	1.35	humid	25.12	20.47	−23.9	0.9
AU‐Tum	1.34	humid	28.98	22.33	−32.4	−16.3
IT‐SRo	1.34	humid	20.78	14.64	−34.7	−13.7
US‐KS2	1.21	humid	40.95	17.2	88.1	24.6
CA‐Man	1.19	humid	21.68	16.62	85.6	33.5
CA‐NS4	1.19	humid	10.51	8.85	45.1	29.7
DE‐Gri	1.18	humid	21.36	18.19	−34.9	−27.8
IT‐MBo	1.18	humid	15.58	13.19	−3.1	−18.4
RU‐Ha1	1.11	humid	7.48	6.4	−16.4	−14.6
FR‐LBr	1.1	humid	15.57	12.29	−30.6	−20.2
US‐Wi6	1.08	humid	14.74	21.4	59.1	86.6
AR‐Vir	1.02	humid	23.03	26.32	−21.5	−20
US‐PFa	1.02	humid	13.32	16.33	46.6	54.7
US‐Syv	1.01	humid	13.98	10.97	15.6	−9.7
RU‐Fyo	0.97	humid	15.41	15.45	−20.1	−26.6
BE‐Bra	0.91	humid	9.3	6.62	−6.8	0
FI‐Hyy	0.87	humid	14.8	10.17	−11.7	−18.9
NL‐Hor	0.84	humid	13.34	12.57	−6.1	−6.8
CH‐Oe1	0.8	humid	28.33	30.06	−30.2	−37.2
BR‐Sa3	0.78	humid	38.47	26.22	31	−21
CZ‐BK2	0.78	humid	20.05	19.45	24.1	21.3
DE‐RuR	0.78	humid	21.85	16.78	37.1	−24.8
BE‐Vie	0.73	humid	16.21	19.69	−22.5	−35.2
CH‐Fru	0.71	humid	28.03	23.37	44.5	−34.8
IT‐Tor	0.63	humid	13.73	13.66	38.6	−34.7

**FIGURE 8:**
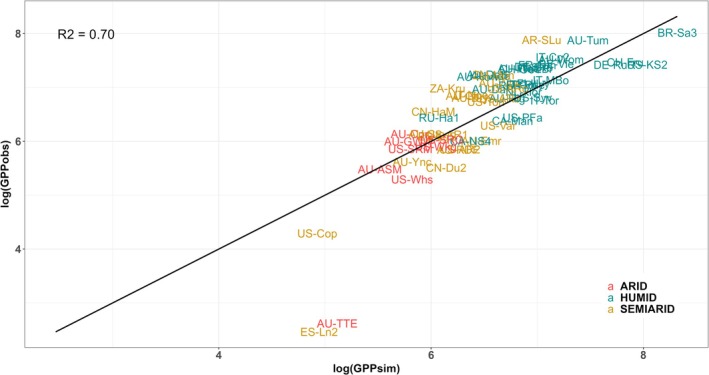
Comparison of simulated and observed total annual gross primary production across multiple sites. Comparison of simulated total annual gross primary production including the new soil‐moisture stress function, with its aridity‐based parameters, and the flux‐derived total annual gross primary production across 67 sites classified by aridity (arid, semi‐arid, humid). Points represent log‐transformed values of GPP observed (log(GPPobs) and simulated (log(GPPsim) at the different sites. Each point shows the multiyear mean of total annual GPP at each site, calculated from years with > 50% data coverage and > 0.8 data quality. The 1:1 line indicates ideal model performance. GPP is in units of gC m^−2^ year^−1^.

Figure 8 illustrates how well the model explains variations in total annual GPP across different sites and aridity classes. It compares observed and simulated total annual GPP—computed by summing daily GPP values, accounting for soil–water stress using the new empirical soil‐moisture stress function, and averaging across multiple years with adequate data coverage (> 50%) and data quality (> 0.8) for each site. Total annual GPP is in units of gC m^−2^ year^−1^.

Across most sites, the model captures a reasonably consistent pattern of GPP over‐ or underestimation across years, suggesting stability in performance (preliminary figures not shown). Consequently, we averaged GPP across years to obtain a multi‐year mean of total annual GPP per site, given that no differences among years were highlighted from preliminary investigations. This approach also ensures data consistency by excluding years with low data coverage and poor data quality.

The majority of sites cluster closely around the 1:1 line, indicating that the model performs well overall after the application of the new function. To further assess the performance of the model, we conducted an additional experiment using site‐specific parameters instead of the aridity‐based approach. As shown in Supplementary Figure 16, this approach yielded a slightly higher R^2^ value (0.85) compared to the aridity‐based parameterisation (0.70, Figure 8). This result confirms that the aridity‐based formulation effectively captures most of the variability while some site‐specific differences remain unaccounted for. While some arid sites show minor overestimations or underestimations, both semi‐arid and humid sites exhibit a slight tendency for the model to underestimate annual GPP. Overall, no clear systematic bias is apparent across aridity classes, and a good fit with the observations is noticeable in the majority of sites analysed.

There are, however, some notable exceptions where the model fails to match observed GPP (Figure 8; Supplementary Figures 6, 7). Specifically, the sub‐daily P model with the new function fails to capture the trends of observed GPP at the semi‐arid site ES‐Ln2 and the arid site AU‐TTE (Supplementary Figures 6, 7), as does the sub‐daily P model with the previous function (Supplementary Figures 9, 10). Furthermore, these two sites are also sites where the MODIS GPP model shows a very large bias, similar to the bias of the sub‐daily P model (Supplementary Figures 12, 13). Both sites have distinct characteristics that likely limit the model's ability to capture the observed GPP. The arid AU‐TTE site experiences highly seasonal rainfall with considerable interannual variability, while the semi‐arid ES‐Ln2 site is a managed ecosystem.

## 4 Discussion

We have developed an empirical function to account for soil‐moisture stress on LUE in the sub‐daily version of the P model (P‐model subDaily v1.0.0). The introduction of an empirical function to account for soil‐moisture stress, previously developed by Stocker et al. (2020) for use with the standard P model (Pv1.0), improved the simulation of GPP by focusing on reducing GPP when soil moisture was below a critical threshold of the β(θ) ratio. This stress function does not perform as well in the sub‐daily P model. This reflects differences in the acclimation timescales of the two models: the standard P model acclimates to daily average conditions, while the sub‐daily P model optimises for noon conditions when GPP responses to soil‐moisture stress are more pronounced. Consequently, a stronger correction is needed in the sub‐daily P model to capture this effect adequately. By incorporating a reduction in the maximum level of the β(θ) ratio with increasing aridity, we have further improved the performance of the sub‐daily P model. However, the comparison with the Stocker et al. (2020) correction shows that the two corrections are not interchangeable.

The performance of the P‐model subDaily v1.0.0 is similar to that of the most recent and improved gap‐filled version of MODIS GPP (MOD17A2HGF v 0.61). MODIS is a widely used product but uses a PFT‐specific parametrisation, whereas the P model makes no distinctions by PFTs. Furthermore, whereas MODIS is empirically based, the P model has a strong theoretical basis in eco‐evolutionary optimality theory, allowing it to take account of the impact of changing CO_2_ on assimilation in a natural way. Thus, our theory‐based and parameter‐sparse model provides an alternative approach that performs as well as the MODIS product.

The application of the new function substantially reduces the overestimation of GPP compared to the original model and to the moisture stress function developed by Stocker et al. (2020) when applied in the sub‐daily model. However, the model does not always capture peaks in GPP shown by the observations; it also overestimates GPP outside the growing season at some sites (e.g., US‐Var). It is difficult to identify the causes of specific mismatches between eddy‐covariance‐derived and simulated GPP on particular days or weeks because such mismatches can have multiple causes. In addition to possible issues with the model itself, there is uncertainty in the partitioning of measured net ecosystem exchange to GPP versus ecosystem respiration (particularly during the non‐growing season) and unavoidable discrepancies between the satellite‐derived pixel data and the footprint of the flux tower (Prentice et al. 2024).

The reduction in the maximum level of LUE with increasing aridity is consistent with the analyses of Fu et al. (2022), which focused on EF. The climatological aridity index (AI) provides a measure of the degree to which water is likely to be limiting (to both EF and LUE) at some time during the growing season. Our findings confirm the proposed hypothesis that the response of GPP to soil moisture varies systematically with climatic aridity, rather than being dependent on the type of vegetation. This pattern reflects different water‐use strategies adopted by plants to optimise carbon assimilation over the whole growing season in the climate to which they are adapted (Manzoni et al. 2011b; Manzoni et al. 2014; Bassiouni, Manzoni, and Vico 2023; Vico et al. 2013; Fu et al. 2022). The fact that there is a limitation on EF and LUE—even during intervals with abundant soil moisture—in more arid climates supports this hypothesis of climate‐adapted water conservation strategies. Moreover, as also noted by Fu et al. (2022) for EF, the slope of β(θ) against θ (*y*/ψ in equation (1)) becomes steeper with increasing aridity. This behaviour results from the values of the exponent of AI in Equations (4) and (5) (0.60 > 0.45), which indicate that *y*/ψ is an increasing function of AI. It implies that, for every value of AI, there is a value of θ at which the associated LUE is maximised, exceeding that of all other β(θ) functions, and that this optimal θ value declines as AI increases.

It is well known that some plants continue to photosynthesise at higher levels of drought stress than others, a behaviour that reflects variability in the strictness of stomatal regulation (Tardieu and Simoneau 1998; McDowell et al. 2008). However, both strict (isohydric) regulation and less strict (anisohydric) regulation can occur within the same community (e.g., Mediavilla and Escudero 2003; Cruz de Souza et al. 2020; Raffelsbauer et al. 2023) and species may show variable regulation over the season and between years (Klein 2014; Konings and Gentine 2017). Thus, although there is some evidence that this behaviour is environmentally controlled (Manzoni et al. 2011b; McDowell 2011; Kumagai and Porporato 2012; Zhou et al. 2014; Konings and Gentine 2017), consistent with our finding that the critical threshold becomes lower as climatological aridity increases, it is likely that plant communities often show a diversity of responses. Our results indicate considerable scatter in both fitted parameters, whose origin and potential adaptive significance would repay more detailed study.

This work was originally designed to improve the performance of the P model and provide a simple algorithm that could have more general utility in land surface modelling. The new approach enables the highly parsimonious and parameter‐sparse sub‐daily P model to be applied globally, across a wide range of aridity and soil moisture conditions, without requiring complex parametrisation or tuning. How best to represent soil moisture in this context is a challenge. We have opted for a minimalist approach, using SPLASH. SPLASH is a single‐bucket model that considers only water that is held between the wilting point and field capacity, and does not account for variation in water holding capacity among soils. The *x‐*intercept of the breakpoint relationship corresponds to the wilting point. We have constrained breakpoint regressions through the origin since little information was lost by doing so. In reality, the permanent wilting point varies across species (Koepke, Kolb, and Adams 2010; Bartlett, Scoffoni, and Sack 2012) but is also strongly affected by soil properties (Czyż and Dexter 2012; Chagas Torres et al. 2021), complexities that we have intentionally ignored to retain the general applicability of our approach. By using a generic soil water balance model, we have also intentionally decoupled AET (computed by SPLASH on the assumption that the ratio AET/PET is proportional to relative soil water content) from GPP, thus disregarding the feedback by which seasonal changes in GPP can influence the seasonal time course of AET and soil moisture. This trade‐off simplifies the derivation of the function and facilitates its implementation in a global modelling framework. However, while ignoring the coupling is a logical starting point since the fully coupled system may show a greater variety of behaviours, consideration of the coupling will be necessary for climate modelling applications.

We have developed an empirical soil‐moisture stress function that not only improves the performance of the P model but is also readily transferable to other modelling contexts. This research, therefore, represents a step towards an empirically well‐founded representation of the interactions between carbon and water cycling, where the next step will involve the interactive coupling of transpiration and GPP in a land‐surface modelling framework. However, we have used a long‐term average of climate parameters to calculate the aridity index (AI). Under a changing climate, AI will change along with changes in vegetation properties such as rooting depth and hydraulic strategy. This poses two practical questions about how to implement our approach in the context of future climate change. First, what is the appropriate timescale at which to update the AI calculation? Second, how will the response to aridity be modified by changes in atmospheric CO_2_? Both questions are likely related to trait plasticity, plant lifespan and the speed and magnitude of climate change. Further research should prioritise addressing these questions to enhance the applicability of our approach in a changing world.

## 5 Conclusions

We have derived a new empirical function to account for the soil moisture effect on the light‐use efficiency of GPP as a function of climatological aridity. The new function provides a constraint on both the maximum level of GPP and the critical soil‐moisture threshold, with increasing climatological aridity. Climatological aridity provides a measure of the degree to which water is likely to be limiting at some time during the growing season. The new formulation is thus consistent with the idea that plants adopt water conservation strategies to optimise assimilation over the whole growing season in the climate to which they are adapted. The new formulation produces an improved simulation of GPP at flux tower sites from arid, semi‐arid and humid regions, both during water‐stressed conditions and during unstressed periods. Although this new function is tested in the context of the existing LUE model (the P model), it is generic and could easily be implemented in other models, including land‐surface schemes.

## Author Contributions

**I. Colin Prentice:** conceptualization, funding acquisition, methodology, writing – review and editing. **Sandy P. Harrison:** conceptualization, funding acquisition, methodology, writing – original draft, writing – review and editing. **Giulia Mengoli:** conceptualization, data curation, formal analysis, methodology, software, writing – original draft, writing – review and editing.

## Conflicts of Interest

The authors declare no Conflicts of Interest.

## Supporting information


Data S1.

## Data Availability

The data and code that support the findings of this study are openly available in Zenodo at https://doi.org/10.5281/zenodo.8018299 and https://doi.org/10.5281/zenodo.8018599 . The code for the SPLASH was obtained from Zenodo at https://doi.org/10.5281/zenodo.376293 and https://bitbucket.org/labprentice/splash/src/master/. Meteorological, satellite and gridded climate datasets were obtained from FLUXNET at https://fluxnet.org/data/fluxnet2015‐dataset/ (FLUXNET2015), Zenodo at http://doi.org/10.5281/zenodo.4392703, the University of East Anglia Climatic Research Unit database at https://catalogue.ceda.ac.uk/uuid/e0b4e1e56c1c4460b796073a31366980/ (CRU TS4.06), and the NASA EOSDIS Land Processes Distributed Active Archive Center at https://doi.org/10.5067/MODIS/MOD17A2HGF.061 (MOD17A2HGFv061).
