# Supplementary material for: The Response of Carbon Uptake to Soil Moisture Stress: Adaptation to Climatic Aridity
Source: Glob Chang Biol. 2025 Mar 10;31(3):e70098. doi: 10.1111/gcb.70098 (PMC11892089; doi:10.1111/gcb.70098)
Supplement: Supplementary file 1 — Data S1. [file GCB-31-e70098-s001.docx]

**The response of carbon uptake to soil moisture stress: adaptation to climatic aridity**

Giulia Mengoli^1,2^, Sandy P. Harrison^2,3^, I. Colin Prentice^1,3^

1: Georgina Mace Centre for the Living Planet, Department of Life Sciences, Imperial College London, Silwood Park Campus, Buckhurst Road, Ascot, SL5 7PY, UK

2: Department of Geography and Environmental Science, School of Archaeology, Geography and Environmental Science (SAGES), University of Reading, Reading, RG6 6AH, UK

3: Ministry of Education Key Laboratory for Earth System Modelling, Department of Earth System Science, Tsinghua University, Beijing 100084, China

*Correspondence to*: Giulia Mengoli ([giulia.mengoli@gmail.com)](mailto:gmengoli@ic.ac.uk))

**Supplementary Information**

This Supplementary Information contains the following tables, figures and text:

Supplementary Table 1. Characteristics of the flux tower sites used in the analysis, giving the unique code for each site (Site ID), latitude, longitude, elevation, calculated aridity index (AI), climate classification, vegetation classification, sampling years (recording period) and reference. The climate type follows Köppen system, where Aw is tropical savanna, Am is tropical monsoon, BSk is cold semi-arid or steppe, BSh is hot semi-arid or steppe, BWh is hot arid desert, Csa is temperate with dry hot summer, Cfa is temperate with no dry season and hot summer, Cfb is temperate with no dry season and warm summer, Cwa is temperate with dry winter and hot summer, Dfc is continental with no dry season and cold summer, Dwb is continental with dry winter and warm summer, Dfb is continental with no dry season and warm summer, and ET is polar tundra. The ecosystem type is based on the International Geosphere–Biosphere Programme (IGBP) definition, where ENF is evergreen needleaf forest, DBF is deciduous broadleaf forest, EBF is evergreen broadleaf forest, MF is mixed forest, WSA is woody savanna, SAV is savanna, CSH is closed shrubland, OSH is open shrubland, and GRA is grassland.

Supplementary Figure 1: Box-plot showing the range of intercept values obtained across all the flux tower sites, grouped by aridity class. The black line is the median value, the box is the interquartile range and the whiskers show the range, with outliers shown as asterisks. The median value is not significantly different from zero.

Supplementary Figure 2: Values of the fitted maximum β(θ) ratio (the ratio of actual flux-derived to modelled well-watered gross primary production) and the critical threshold value of soil moisture for all 67 sites used in the analysis, where the intercept is assumed to be zero. The β(θ) ratio and the soil water content (swc) are both unitless. Note that the scale above 1 has been compressed for visualization purposes.

Supplementary Figure 3: Values of the fitted maximum β(θ) ratio (the ratio of actual flux-derived to modelled well-watered gross primary production) and the critical threshold value of soil moisture for all 67 sites used in the analysis, where the intercept is assumed to be zero (green line) or not fixed (red line). The β(θ) ratio and the soil water content (swc) are both unitless. Note that the scale above 1 has been compressed for visualization purposes.

Supplementary Figure 4: The fitted non-linear regression model of the maximum level (top) and the critical threshold (bottom) of the β(θ) ratio (the ratio of observed to predicted gross primary production) against the aridity index, where the sites are classified according to vegetation type and precipitation phase.

Supplementary Figure 5: The fitted non-linear regression model of the maximum level (top) and the critical threshold (bottom) of the β(θ) ratio (the ratio of observed to predicted gross primary production) against the aridity index, where the sites are classified according to vegetation type and precipitation concentration.

Supplementary Figure 6: The impact of the application of the new soil moisture stress function on simulated gross primary production (GPP_new_) at flux tower sites classified as arid (aridity index, AI >5). The new model is compared to the simulated level of GPP under well-watered conditions (GPP_ww_) and to flux-derived values (GPP_obs_).

Supplementary Figure 7: The impact of the application of the new soil moisture stress function on simulated gross primary production (GPP_new_) at flux tower sites classified as semi-arid (aridity index, AI between 2 and 5). The new model is compared to the simulated level of GPP under well-watered conditions (GPP_ww_) and to flux-derived values (GPP_obs_).

Supplementary Figure 8: The impact of the application of the new soil moisture stress function on simulated gross primary production (GPP_new_) at flux tower sites classified as humid (aridity index, AI <2). The new model is compared to the simulated level of GPP under well-watered conditions (GPP_ww_) and to flux-derived values (GPP_obs_).

Supplementary Figure 9: Comparison of simulated gross primary production including the new soil-moisture stress function (GPP_new_) and the original stress function (GPP_v1.0_) from Stocker et al. (2020) against flux-derived values (GPP_obs_) at flux tower sites classified as arid (aridity index, AI > 5).

Supplementary Figure 10: Comparison of simulated gross primary production including the new soil-moisture stress function (GPP_new_) and the original stress function (GPP_v1.0_) from Stocker et al. (2020) against flux-derived values (GPP_obs_) at flux tower sites classified as semi-arid (aridity index, AI = between 2 and 5).

Supplementary Figure 11: Comparison of simulated gross primary production including the new soil-moisture stress function (GPP_new_) and the original stress function (GPP_v1.0_) from Stocker et al. (2020) against flux-derived values (GPP_obs_) at flux tower sites classified as humid (aridity index, AI < 2).

Supplementary Figure 12: Comparison of simulated gross primary production including the new soil-moisture stress function (GPPnew) and the gross primary production simulated by MOD17A2HGF v0.61 (GPP_MODIS_) against flux-derived values (GPPobs) at flux tower sites classified as arid (aridity index, AI > 5).

Supplementary Figure 13: Comparison of simulated gross primary production including the new soil-moisture stress function (GPPnew) and the gross primary production simulated by MOD17A2HGF v0.61 (GPP_MODIS_) against flux-derived values (GPPobs) at flux tower sites classified as semi-arid (aridity index, AI = between 2 and 5).

Supplementary Figure 14: Comparison of simulated gross primary production including the new soil-moisture stress function (GPPnew) and the gross primary production simulated by MOD17A2HGF v0.61 (GPP_MODIS_) against flux-derived values (GPPobs) at flux tower sites classified as humid (aridity index, AI < 2).

Supplementary text: Potential explanations for the poor model performance at ES-Ln2 and AU-TTE.

**Supplementary Table 1**. Characteristics of the flux tower sites used in the analysis, giving the unique code for each site (Site ID), latitude, longitude, elevation, calculated aridity index (AI), climate classification, vegetation classification, sampling years (recording period) and reference. The climate type follows Köppen system, where Aw is tropical savanna, Am is tropical monsoon, BSk is cold semi-arid or steppe, BSh is hot semi-arid or steppe, BWh is hot arid desert, Csa is temperate with dry hot summer, Cfa is temperate with no dry season and hot summer, Cfb is temperate with no dry season and warm summer, Cwa is temperate with dry winter and hot summer, Dfc is continental with no dry season and cold summer, Dwb is continental with dry winter and warm summer, Dfb is continental with no dry season and warm summer, and ET is polar tundra. The ecosystem type is based on the International Geosphere–Biosphere Programme (IGBP) definition, where ENF is evergreen needleaf forest, DBF is deciduous broadleaf forest, EBF is evergreen broadleaf forest, MF is mixed forest, WSA is woody savanna, SAV is savanna, CSH is closed shrubland, OSH is open shrubland, and GRA is grassland.

| **Site ID** | **Latitude**  **(°)** | **Longitude**  **(°)** | **Elevation (m)** | **AI** | **Climate** | **IGBP** | **Recording period** | **Reference** |
| --- | --- | --- | --- | --- | --- | --- | --- | --- |
| AR-SLu | -33.46 | -66.46 | 507 | 2.89 | BSk | MF | 2009-2011 | Ulke et al. (2015) |
| AR-Vir | -28.24 | -56.19 | 104 | 1.02 | Cfa | ENF | 2009-2012 | Posse et al. (2016) |
| AU-Ade | -13.08 | 131.12 | 79 | 1.55 | Aw | WSA | 2007-2009 | Beringer et al. (2011b) |
| AU-ASM | -22.28 | 133.25 | 605 | 6.97 | BSh | SAV | 2010-2013 | Cleverly et al. (2013) |
| AU-Cpr | -34.00 | 140.59 | 60 | 6.36 | BSk | SAV | 2010-2014 | Meyer et al. (2015) |
| AU-DaP | -14.06 | 131.32 | 69 | 1.80 | Aw | GRA | 2007-2013 | Beringer et al. (2011a) |
| AU-DaS | -14.16 | 131.39 | 79 | 1.81 | Aw | SAV | 2010-2014 | Hutley et al. (2011) |
| AU-Dry | -15.26 | 132.37 | 175 | 2.32 | Aw | SAV | 2008-2014 | Cernusak et al. (2011) |
| AU-Emr | -23.86 | 148.47 | 175 | 3.08 | BSh | GRA | 2011-2013 | Schroder et al. (2014) |
| AU-Gin | -31.38 | 115.71 | 50 | 2.93 | Csa | WSA | 2012-2014 | Beringer et al. (2016) |
| AU-GWW | -30.19 | 120.65 | 446 | 5.75 | BSh | SAV | 2013-2014 | Prober et al. (2012) |
| AU-How | -12.49 | 131.15 | 35 | 1.46 | Aw | WSA | 2003-2008 | Beringer et al. (2007) |
| AU-Lox | -34.47 | 140.66 | 43 | 6.32 | BSk | DBF | 2008-2009 | Stevens et al. (2011) |
| AU-RDF | -14.56 | 132.48 | 181 | 2.16 | Aw | WSA | 2011-2013 | Bristow et al. (2016) |
| AU-Rig | -36.65 | 145.58 | 151 | 1.81 | Cfb | GRA | 2011-2014 | Beringer et al. (2016) |
| AU-Stp | -17.15 | 133.35 | 229 | 3.71 | BSh | GRA | 2010-2014 | Beringer et al. (2011a) |
| AU-TTE | -22.29 | 133.64 | 551 | 7.17 | BWh | GRA | 2012-2013 | Cleverly et al. (2016) |
| AU-Tum | -35.66 | 148.15 | 1238 | 1.34 | Cfb | EBF | 2007-2014 | Leuning et al. (2005) |
| AU-Wac | -37.43 | 145.19 | 732 | 1.69 | Cfb | EBF | 2005-2008 | Kilinc et al. (2013) |
| AU-Whr | -36.67 | 145.03 | 144 | 2.39 | Cfb | EBF | 2011-2014 | McHugh et al. (2017) |
| AU-Wom | -37.42 | 144.09 | 700 | 1.75 | Cfb | EBF | 2010-2012 | Hinko-Najera et al. (2017) |
| AU-Ync | -34.99 | 146.29 | 127 | 3.96 | BSk | GRA | 2012-2014 | Yee et al. (2015) |
| BE-Bra | 51.31 | 4.52 | 16 | 0.91 | Cfb | MF | 2007-2014 | Carrara et al. (2004) |
| BE-Vie | 50.30 | 6.00 | 486 | 0.73 | Cfb | MF | 2010-2014 | Aubinet et al. (2001) |
| BR-Sa3 | -3.02 | -54.97 | 172 | 0.78 | Am | EBF | 2001-2004 | Wick et al. (2005) |
| CA-Man | 55.88 | -98.48 | 261 | 1.19 | Dfc | ENF | 2003-2008 | Dunn et al. (2007) |
| CA-NS4 | 55.91 | -98.38 | 252 | 1.19 | Dfc | ENF | 2002-2005 | Chu et al. (2021) |
| CA-SF3 | 54.09 | -106.01 | 544 | 1.41 | Dfc | OSH | 2002-2006 | Chu et al. (2021) |
| CH-Fru | 47.12 | 8.54 | 972 | 0.71 | Cfb | GRA | 2007-2014 | Imer et al. (2013) |
| CH-Oe1 | 47.29 | 7.73 | 454 | 0.80 | Cfb | GRA | 2003-2008 | Ammann et al. (2009) |
| CN-Du2 | 42.05 | 116.28 | 1321 | 2.70 | Dwb | GRA | 2006-2008 | Chen et al. (2009) |
| CN-HaM | 37.37 | 101.18 | 4032 | 2.34 | ET | GRA | 2002-2004 | Kato et al. (2006) |
| CZ-BK2 | 49.49 | 18.54 | 844 | 0.78 | Dfb | GRA | 2004-2006 | NA |
| DE-Gri | 50.95 | 13.51 | 380 | 1.18 | Cfb | GRA | 2010-2014 | Prescher et al. (2010) |
| DE-RuR | 50.62 | 6.30 | 514 | 0.78 | Cfb | GRA | 2011-2014 | Post et al. (2015) |
| ES-LgS | 37.10 | -2.97 | 2271 | 2.88 | Csa | OSH | 2007-2009 | Reverter et al. (2010) |
| ES-Ln2 | 36.97 | -3.48 | 2215 | 3.84 | Csa | OSH | 2009-2009 | Serrano-Ortiz et al. (2011) |
| FI-Hyy | 61.85 | 24.29 | 177 | 0.87 | Dfc | ENF | 2010-2014 | Suni et al. (2003) |
| FR-Fon | 48.48 | 2.78 | 93 | 1.39 | Cfb | DBF | 2007-2013 | Delpierre et al. (2015) |
| FR-LBr | 44.72 | -0.77 | 63 | 1.10 | Cfb | ENF | 2003-2008 | Berbigier et al. (2001) |
| FR-Pue | 43.74 | 3.60 | 269 | 1.57 | Csa | EBF | 2003-2007 | Rambal et al. (2004) |
| IT-Col | 41.85 | 13.59 | 1549 | 1.35 | Cfa | DBF | 2007-2014 | Valentini et al. (1996) |
| IT-Cp2 | 41.70 | 12.36 | 3 | 1.73 | Csa | EBF | 2012-2014 | Fares et al. (2014) |
| IT-MBo | 46.01 | 11.05 | 1549 | 1.18 | Dfb | GRA | 2007-2013 | Marcolla et al. (2011) |
| IT-Noe | 40.61 | 8.15 | 29 | 2.26 | Csa | CSH | 2004-2008 | Papale et al. (2014) |
| IT-SRo | 43.73 | 10.28 | 3 | 1.34 | Csa | ENF | 2003-2008 | Chiesi et al. (2005) |
| IT-Tor | 45.84 | 7.58 | 2164 | 0.63 | Dfc | GRA | 2008-2014 | Galvagno et al. (2013) |
| NL-Hor | 52.24 | 5.07 | 1 | 0.84 | Cfb | GRA | 2006-2011 | Jacobs et al. (2007) |
| RU-Fyo | 56.46 | 32.92 | 268 | 0.97 | Dfb | ENF | 2010-2014 | Kurbatova et al. (2008) |
| RU-Ha1 | 54.73 | 90.00 | 453 | 1.11 | Dfc | GRA | 2002-2004 | Marchesini et al. (2007) |
| US-AR1 | 36.43 | -99.42 | 612 | 2.49 | Cfa | GRA | 2009-2012 | Chu et al. (2021) |
| US-AR2 | 36.64 | -99.60 | 645 | 2.61 | BSk | GRA | 2009-2012 | Chu et al. (2021) |
| US-ARb | 35.55 | -98.04 | 423 | 2.04 | Cfa | GRA | 2005-2006 | Fischer et al. (2012) |
| US-ARc | 35.55 | -98.04 | 423 | 2.04 | Cfa | GRA | 2005-2006 | Fischer et al. (2012) |
| US-Cop | 38.09 | -109.39 | 1903 | 3.99 | BSk | GRA | 2002-2007 | Bowling et al. (2010) |
| US-KS2 | 28.61 | -80.67 | 2 | 1.21 | Cfa | CSH | 2003-2006 | Powell et al. (2006) |
| US-PFa | 45.95 | -90.27 | 471 | 1.02 | Dfb | MF | 2010-2014 | Desai et al. (2015) |
| US-SRG | 31.79 | -110.83 | 1293 | 5.08 | BSk | GRA | 2008-2014 | Scott et al. (2015a) |
| US-SRM | 31.82 | -110.87 | 1113 | 5.02 | BSk | WSA | 2008-2014 | Scott et al. (2009) |
| US-Syv | 46.24 | -89.35 | 544 | 1.01 | Dfb | MF | 2010-2014 | Desai et al. (2005) |
| US-Ton | 38.43 | -120.97 | 174 | 2.23 | Csa | WSA | 2003-2007 | Baldocchi et al. (2010) |
| US-Var | 38.41 | -120.95 | 166 | 2.22 | Csa | GRA | 2008-2014 | Ma et al. (2007) |
| US-Whs | 31.74 | -110.05 | 1372 | 5.89 | BSk | OSH | 2007-2014 | Scott et al. (2015a) |
| US-Wi6 | 46.62 | -91.30 | 354 | 1.08 | Dfb | OSH | 2002-2003 | Noormets et al. (2007) |
| US-Wkg | 31.74 | -109.94 | 1515 | 6.34 | BSk | GRA | 2007-2014 | Scott et al. (2010) |
| ZA-Kru | -25.02 | 31.50 | 357 | 2.69 | Cwa | SAV | 2005-2010 | Archibald et al. (2009) |
| ZM-Mon | -15.44 | 23.25 | 1087 | 2.18 | Aw | DBF | 2003-2008 | Merbold et al. (2009) |

**References for Table 1**

Ammann, C., Spirig, C., Leifeld, J., and Neftel, A.: Assessment of the nitrogen and carbon budget of two managed temperate grassland fields, Agric. Ecosyst. Environ., 133, 150–162, 2009.

Archibald, S.A., Kirton, A., van der Merwe, M.R., Scholes, R.J., Williams, C.A., and Hanan, N.: Drivers of inter-annual variability in Net Ecosystem Exchange in a semi-arid savanna ecosystem, South Africa, Biogeosci., 6, 251–266, 2009.

Aubinet, M., Chermanne, B., Vandenhaute, M., Longdoz, B., Yernaux, M., and Laitat, E.: Long term carbon dioxide exchange above a mixed forest in the Belgian Ardennes, Agric. For. Meteorol., 108, 293–315, 2001.

Baldocchi, D., Chen, Q., Chen, X., Ma, S., Miller, G., Ryu, Y., Xiao, J., Wenk, R., Battles, J.: The dynamics of energy, water, and carbon fluxes in a blue oak (*Quercus douglasii*) savanna in California, Ecosyst. Funct. Savannas, 132, 135-151, 2010

Berbigier, P., Bonnefond, J.-M. and Mellmann, P.: CO_2_ and water vapour fluxes for 2 years above Euroflux forest site, Agric. For. Meteorol., 108, 183–197, 2001.

Beringer, J., Hutley, L.B., Tapper, N.J., and Cernusak, L.A.: Savanna fires and their impact on net ecosystem productivity in North Australia, Glob. Chang. Biol., 13, 990–1004, 2007.

Beringer, J., Hutley, L.B., Hacker, J.M., Neininger, B., and Tha Paw U.K.: Patterns and processes of carbon, water and energy cycles across northern Australian landscapes: From point to region, Agric. For. Meteorol., 151, 1409–1416, 2011a.

Beringer, J., Hacker, J., Hutley, L.B., Leuning, R., Arndt, S.K., Amiri, R., Bannehr, L., Cernusak, L., Grover, S., Hensley, C., Hocking, D., Isaac, P., Jamali, H., Kanniah, K., Livesley, S., Neininger, B., Tha Paw U.K., Sea, W., Straten, D., Tapper, N., Weinmann, R., Wood, S., and Zegelin, S.: SPECIAL—Savanna patterns of energy and carbon integrated across the landscape, Bull. Am. Meteorol. Soc., 92, 1467–1485, 2011b.

Beringer, J., Hutley, L.B., McHugh, I., Arndt, S.K., Campbell, D., Cleugh, H.A., Cleverly, J., Resco de Dios, V., Eamus, D., Evans, B., Ewenz, C., Grace, P., Griebel, A., Haverd, V., Hinko-Najera, N., Huete, A., Isaac, P., Kanniah, K., Leuning, R., Liddell, M.J., Macfarlane, C., Meyer, W., Moore, C., Pendall, E., Phillips, A., Phillips, R.L., Prober, S.M., Restrepo-Coupe, N., Rutledge, S., Schroder, I., Silberstein, R., Southall, P., Yee, M.S., Tapper, N.J., van Gorsel, E., Vote, C., Walker, J., and Wardlaw, T.: An introduction to the Australian and New Zealand flux tower network – OzFlux, Biogeosci., 13, 5895–5916, https://doi.org/10.5194/bg-13-5895-2016, 2016.

Bowling, D.R., Bethers‐Marchetti, S., Lunch, C.K., Grote, E.E., and Belnap. J.: Carbon, water, and energy fluxes in a semiarid cold desert grassland during and following multiyear drought, J. Geophys. Res., 115, G04026, doi:10.1029/2010JG001322, 2010.

Bristow, M., Hutley, L.B., Beringer, J., Livesley, S.J., Edwards, A.C., and Arndt, S. K.: Quantifying the relative importance of greenhouse gas emissions from current and future savanna land use change across northern Australia, Biogeosci., 13, 6285–6303, 2016.

Carrara, A., Janssens, I.A., Curiel Yuste, J., and Ceulemans, R.: Seasonal changes in photosynthesis, respiration and NEE of a mixed temperate forest, Agric. For. Meteorol., 126, 15–31, 2004.

Cernusak, L. A., Hutley, L.B., Beringer, J., Holtum, J.A.M. and Turner, B.L.: Photosynthetic physiology of eucalypts along a sub-continental rainfall gradient in northern Australia, Agric. For. Meteorol., 151, 1462–1470, 2011.

Chen, S., Chen, J., Lin, G., Zhang, W., Miao, H., Wei, L., Huang, J., and Han, X.: Energy balance and partition in Inner Mongolia steppe ecosystems with different land use types, Agric. For. Meteorol., 149, 1800–1809, 2009.

Chiesi, M., Maselli, F., Bindi, M., Fibbi, L., Cherubini, P., Arlotta, E., Tirone, G., Matteucci, G., and Seufert, G.: Modelling carbon budget of Mediterranean forests using ground and remote sensing measurements, Agric. For. Meteorol., 135, 22–34, 2005.

Chu, H., Luo, X., Ouyang, Z., Chan, W.S., Dengel, S., Biraud, S.C., Torn, M.S., Metzger, S., Kumar, J., Arain, M.A., Arkebauer, T.J., Baldocchi, D., Bernacchi, C., Billesbach, D., Black, T.A., Blanken, P.D., Bohrer, G., Bracho, R., Brown, S., Brunsell, N.A., Chen, J., Chen, X., Clark, K., Desai, A.R., Duman, T., Durden, D., Fares, S., Forbrich, I., Gamon, J. A., Gough, C.M., Griffis, T., Helbig, M., Hollinger, D., Humphreys, E., Ikawa, H., Iwata, H., Ju, Y., Knowles, J.F., Knox, S.H., Kobayashi, H., Kolb, T., Law, B., Lee, X., Litvak, M., Liu, H., Munger, J.W., Noormets, A., Novick, K., Oberbauer, S.F., Oechel, W., Oikawa, P., Papuga, S.A., Pendall, E., Prajapati, P., Prueger, J., Quinton, W.L., Richardson, A.D., Russell, E.S., Scott, R.L., Starr, G., Staebler, R., Stoy, P.C., Stuart-Haëntjens, E., Sonnentag, O., Sullivan, R.C., Suyker, A., Ueyama, M., Vargas, R., Wood, J.D., and Zona, D.: Representativeness of eddy-covariance flux footprints for areas surrounding Ameriflux sites, Agric. For. Met., 301-30, 108350, [doi.org/10.1016/J.AGRFORMET.2021.108350](https://doi.org/10.1016/J.AGRFORMET.2021.108350), 2021.

Cleverly, J., Boulain, N., Villalobos-Vega, R., Grant, N., Faux, R., Wood, C., Cook, P.G., Yu, Q., Leigh, A., and Eamus, D.: Dynamics of component carbon fluxes in a semi-arid Acacia woodland, central Australia, J. Geophys. Res. Biogeosci., 118, 1168–1185, 2013.

Cleverly, J., Eamus, D., Restrepo Coupe, N., Chen, C., Maes, W., Li, L., Faux, R., Santini, N.S., Rumman, R., Yu, Q., and Huete, A.: Soil moisture controls on phenology and productivity in a semi-arid critical zone, Sci. Total Envir., 568, 1227-1237, doi: 10.1016/j.scittotenv.2016.05.14, 2016.

Delpierre, N., Berveiller, D., Granda, E., and Dufrêne, E.: Wood phenology, not carbon input, controls the interannual variability of wood growth in a temperate oak forest, New Phytol., 210, 459–470, 2016.

Desai, A.R., Bolstad, P.V., Cook, B.D., Davis, K.J., and Carey, E.V.: Comparing net ecosystem exchange of carbon dioxide between an old-growth and mature forest in the Upper Midwest, USA, Agric. Forest Met., 128, 33-55, 2005.

Desai, A.R., Xu, K., Tian, H., Weishampel, P., Thom, J., Baumann, D., Andrews, A.E., Cook, B.D., King, J.Y., and Kolka, R.: Landscape-level terrestrial methane flux observed from a very tall tower, Agric. Forest Met., 201, 61-75, 2015.

Dunn, A.L., Barford, C.C., Wofsy, S.C., Goulden, M.L., and Daube, B.C.: A long-term record of carbon exchange in a boreal black spruce forest: Means, responses to interannual variability, and decadal trends, Glob. Change Biol., 13, 577-590, 2007.

Fares, S., Savi, F., Muller, J., Matteucci, G., and Paoletti, E.: Simultaneous measurements of above and below canopy ozone fluxes help partitioning ozone deposition between its various sinks in a Mediterranean Oak Forest, Agric. For. Meteorol., 198–199, 181–191, 2014.

Fischer, M.L., Torn, M.S., Billesbach, D.P., Doyle, G., Northup, B., and Biraud, S.C.: Carbon, water, and heat flux responses to experimental burning and drought in a tallgrass prairie, Agric. For. Meteorol., 166-167, 169-174, 2012.

Galvagno, M., Wohlfahrt, G., Cremonese, E., Rossini, M., Colombo, R., Filippa, G., Julitta, T., Manca, G., Siniscalco, C., di Cella, U.M., and Migliavacca, M.: Phenology and carbon dioxide source/sink strength of a subalpine grassland in response to an exceptionally short snow season, Environ. Res. Lett., 8, 025008, 2013.

Hinko-Najera, N., Isaac, P., Beringer, J., van Gorsel, E., Ewenz, C., McHugh, I., Exbrayat, J.-F., Livesley, S. J., and Arndt, S. K.: Net ecosystem carbon exchange of a dry temperate eucalypt forest, Biogeosci., 14, 3781–3800, https://doi.org/10.5194/bg-14-3781-2017, 2017.

Hutley, L. B., Beringer, J., Isaac, P.R., Hacker, J.M., and Cernusak, L.A.: A sub-continental scale living laboratory: Spatial patterns of savanna vegetation over a rainfall gradient in northern Australia, Agric. For. Meteorol., 151, 1417–1428, 2011.

Imer, D., Merbold, L., Eugster, W., and Buchmann, N.: Temporal and spatial variations of soil CO_2_, CH_4_ and N_2_O fluxes at three differently managed grasslands, Biogeosci., 10, 5931–5945, 2013.

Jacobs, C.M.J., Jacobs, A.F.G., Bosveld, F.C., Hendriks, D.M.D., Hensen, A., Kroon, P.S., Moors, E.J., Nol, L., Schrier-Uijl, A., and Veenendaal, E.M.: Variability of annual CO_2_ exchange from Dutch grasslands, Biogeosci., 4, 803–816, 2007.

Kato, T., Tang, Y., Gu, S., Hirota, M., Du, M., Li, Y., and Zhao, X.: Temperature and biomass influences on interannual changes in CO2 exchange in an alpine meadow on the Qinghai-Tibetan Plateau, Glob. Chang. Biol., 12, 1285–1298, 2006.

Kilinc, M., Beringer, J., Hutley, L.B., Tapper, N.J., and McGuire, D. A.: Carbon and water exchange of the world’s tallest angiosperm forest, Agric. For. Meteorol., 182–183, 215–224, 2013.

Kurbatova, J., Li, C., Varlagin, A., Xiao, X., and Vygodskaya, N.: Modeling carbon dynamics in two adjacent spruce forests with different soil conditions in Russia, Biogeosci., 5, 969–980, 2008.

Leuning, R., Cleugh, H.A., Zegelin, S.J., and Hughes, D.: Carbon and water fluxes over a temperate *Eucalyptus* forest and a tropical wet/dry savanna in Australia: measurements and comparison with MODIS remote sensing estimates, Agric. For. Meteorol., 129, 151–173, 2005.

Ma, S., Baldocchi, D.D., Xu, L., and Hehn, T.: Inter-annual variability in carbon dioxide exchange of an oak/grass savanna and open grassland in California, Agric. For. Meteorol., 147, 157-171, 2007

Marchesini, L.B., Papale, D., Reichstein, M., Vuichard, N., Tchebakova, N., and Valentini, R.: Carbon balance assessment of a natural steppe of southern Siberia by multiple constraint approach, Biogeosci., 4, 581-595, 2007.

Marcolla, B., Cescatti, A., Manca, G., Zorer, R., Cavagna, M., Fiora, A., Gianelle, D., Rodeghiero, M., Sottocornola, M., and Zampedri, R.: Climatic controls and ecosystem responses drive the inter-annual variability of the net ecosystem exchange of an alpine meadow, Agric. For. Meteorol., 151, 1233–1243, 2011.

McHugh, I.D., Beringer, J., Cunningham, S.C., Baker, P.J., Cavagnaro, T.R., MacNally, R., and Thompson, R. M.: Interactions between nocturnal turbulent flux, storage and advection at an “ideal” eucalypt woodland site, Biogeosci., 14, 3027-3050, 2017.

Merbold, L., Ardö, J., Arneth, A., Scholes, R.J., Nouvellon, Y., de Grandcourt, A., Archibald, S., Bonnefond, J.M., Boulain, N., Brueggemann, N., Bruemmer, C., Cappelaere, B., Ceschia, E., El-Khidir, H.A.M., El-Tahir, B.A., Falk, U., Lloyd, J., Kergoat, L., Dantec, V.L., Mougin, E., Muchinda, M., Mukelabai, M.M., Ramier, D., Roupsard, O., Timouk, F., Veenendaal, E.M., and Kutsch, W. L.: Precipitation as driver of carbon fluxes in 11 African ecosystems, Biogeosci., 6, 1027–1041, 2009.

Meyer, W. S., Kondrlovà, E., and Koerber, G.R.: Evaporation of perennial semi-arid woodland in southeastern Australia is adapted for irregular but common dry periods, Hydrol. Process., 29, 3714–3726, 2015.

Noormets, A., Chen, J., and Crow, T.R.: Age-dependent changes in ecosystem carbon fluxes in managed forests in northern Wisconsin, USA, Ecosystems, 10, 187-203, 2007.

Papale, D., Migliavacca, M., Cremonese E., Cescatti, A., et al.: Carbon, Water and Energy Fluxes of Terrestrial Ecosystems in Italy, in "The Greenhouse Gas Balance of Italy" edited by R. Valentini and F. Miglietta, Springer-Verlag Berlin Heidelberg, doi: 10.1007/978-3-642-32424-6_2, 2015

Posse, G., Lewczuk, N., Richter, K., and Cristiano, P.: Carbon and water vapor balance in a subtropical pine plantation, iForest, 9, 736–742, 2016.

Post, H., Hendricks Franssen, H.J., Graf, A., Schmidt, M., and Vereecken, H.: Uncertainty analysis of eddy covariance CO_2_ flux measurements for different EC tower distances using an extended two-tower approach, Biogeosci., 12, 1205–1221, 2015.

Powell, T.L., Bracho, R., Li, J., Dore, S., Hinkle, C.R., and Drake, B.G.: Environmental controls over net ecosystem carbon exchange of scrub oak in central Florida, Agric. Forest Meteorol., 141, 19-34, https://doi.org/10.1016/j.agrformet.2006.09.002, 2006.

Prescher, A.-K., Grünwald, T., and Bernhofer, C: Land use regulates carbon budgets in eastern Germany: From NEE to NBP. Agric. For. Meteorol., 150, 1016-1025, 2010.

Prober, S. M., Thiele, K. R., Rundel, P.W., Yates, C. J., Berry, S. L., Byrne, M., Christidis, L., Gosper, C. R., Grierson, P. F., Lemson, K., Lyons, T., Macfarlane, C., O’Connor, M. H., Scott, J. K., Standish, R. J., Stock, W. D., van Etten, E. J., Wardell-Johnson, G. W., and Watson, A.: Facilitating adaptation of biodiversity to climate change: A conceptual framework applied to the world’s largest Mediterranean-climate woodland, Clim. Change, 110, 227–248, https://doi.org/10.1007/s10584-011-0092-y, 2012.

Rambal, S., Joffre, R., Ourcival, J.M., Cavender-Bares, J., and Rocheteau, A.: The growth respiration component in eddy CO_2_ flux from a *Quercus ilex* mediterranean forest, Glob. Chang. Biol., 10, 1460–1469, 2004.

Reverter, B.R., Sánchez-Cañete, E.P., Resco, V., Serrano-Ortiz, P., Oyonarte, C., and Kowalski, A.S.: Analyzing the major drivers of NEE in a Mediterranean alpine shrubland, Biogeosci., 7, 2601–2611, 2010.

Schroder, I., Kuske, T., and Zegelin, S.: Eddy covariance dataset for Arcturus (2011-2013), Geoscience Australia, Canberra, doi:102.100.100/14249, 2014.

Scott, R.L., Biederman, J.A., Hamerlynck, E.P., and Barron-Gafford, G.A.: The carbon balance pivot point of southwestern U.S. semiarid ecosystems: Insights from the 21st century drought, J. Geophys. Res. Biogeosci., 120, 2612-2624, 2015a.

Scott, R.L., Hamerlynck, E.P., Jenerette, G.D., Moran, M.S., and Barron-Gafford, G.: Carbon dioxide exchange in a semidesert grassland through drought-induced vegetation change, J. Geophys. Res. Biogeosci., 115, G03026, doi:[10.1029/2010JG001348](https://doi.org/10.1029/2010JG001348), 2010.

Scott, R.L., Jenerette, G.D., Potts, D.L., and Huxman, T.E.: Effects of seasonal drought on net carbon dioxide exchange from a woody-plant-encroached semiarid grassland, J. Geophys. Res. Biogeosci., 114, G04004, doi:[10.1029/2008JG000900](https://doi.org/10.1029/2008JG000900), 2009.

Serrano-Ortiz, P., Marañón-Jiménez, S., Reverter, B.R., Sánchez-Cañete, E.P., Castro, J., Zamora, R., and Kowalski, A.S.: Post-fire salvage logging reduces carbon sequestration in Mediterranean coniferous forest, Forest Ecol. Man., 262, 2287-2296, https://doi.org/10.1016/j.foreco.2011.08.023, 2011

Stevens, R.M., Ewenz, C.M., Grigson, G., and Conner, S.M.: Water use by an irrigated almond orchard, Irrig. Sci., 30, 189–200, 2012.

Suni, T., Rinne, J., Reissell, A., Altimir, N., Keronen, P., Rannik, Ü., Maso, M.D., Kulmala, M., and Vesala, T.: Long-term measurements of surface fluxes above a Scots pine forest in Hyytiälä, southern Finland, 1996–2001, Boreal Environ. Res., 8, 287–301, 2003.

Ulke, A.G., Gattinoni, N.N., and Posse, G.: Analysis and modelling of turbulent fluxes in two different ecosystems in Argentina, Int. J. Environ. Pollut., 58, 52–62, 2015.

Valentini, R., De Angelis, P., Matteucci, G., Monaco, R., Dore, S., and Mugnozza, G. E. S.: Seasonal net carbon dioxide exchange of a beech forest with the atmosphere, Glob. Chang. Biol., 2, 199–207, 1996.

Wick, B., Veldkamp, E., De Mello, W.Z., Keller, M., and Crill, P.: Nitrous oxide fluxes and nitrogen cycling along a pasture chronosequence in central Amazonia, Brazil, Biogeosci., 2, 175-187, 2005.

Yee, M.S., Pauwels, V.R.N., Daly, E., Beringer, J., Rüdiger, C., McCabe, M.F., and Walker, J.P.: A comparison of optical and microwave scintillometers with eddy covariance derived surface heat fluxes, Agric. For. Meteorol., 213, 226–239, 2015.


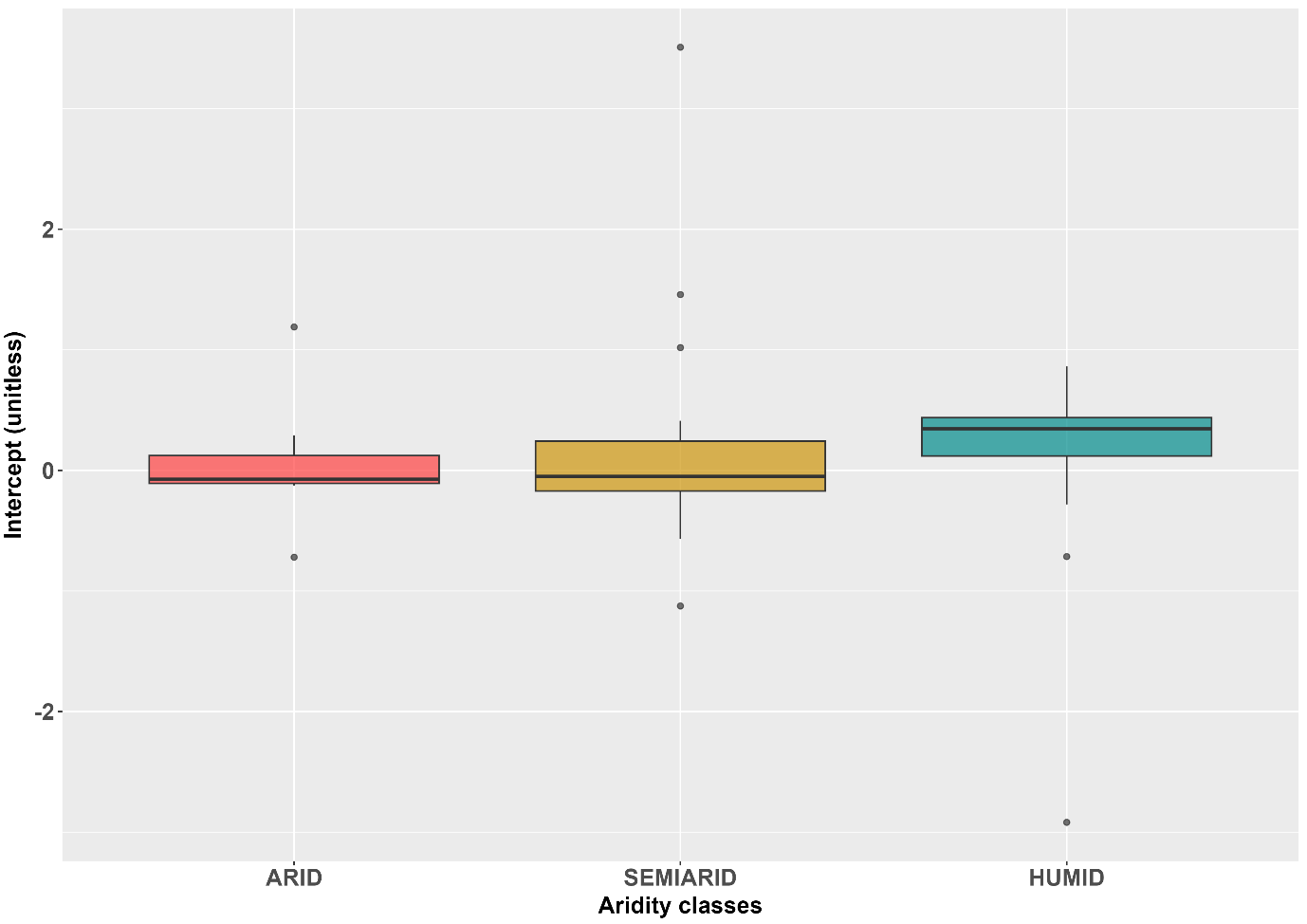


**Supplementary Figure 1**: Box-plot showing the range of intercept values obtained across all the flux tower sites, grouped by aridity class. The black line is the median value, the box is the interquartile range and the whiskers show the range, with outliers shown as asterisks. The median value is not significantly different from zero.


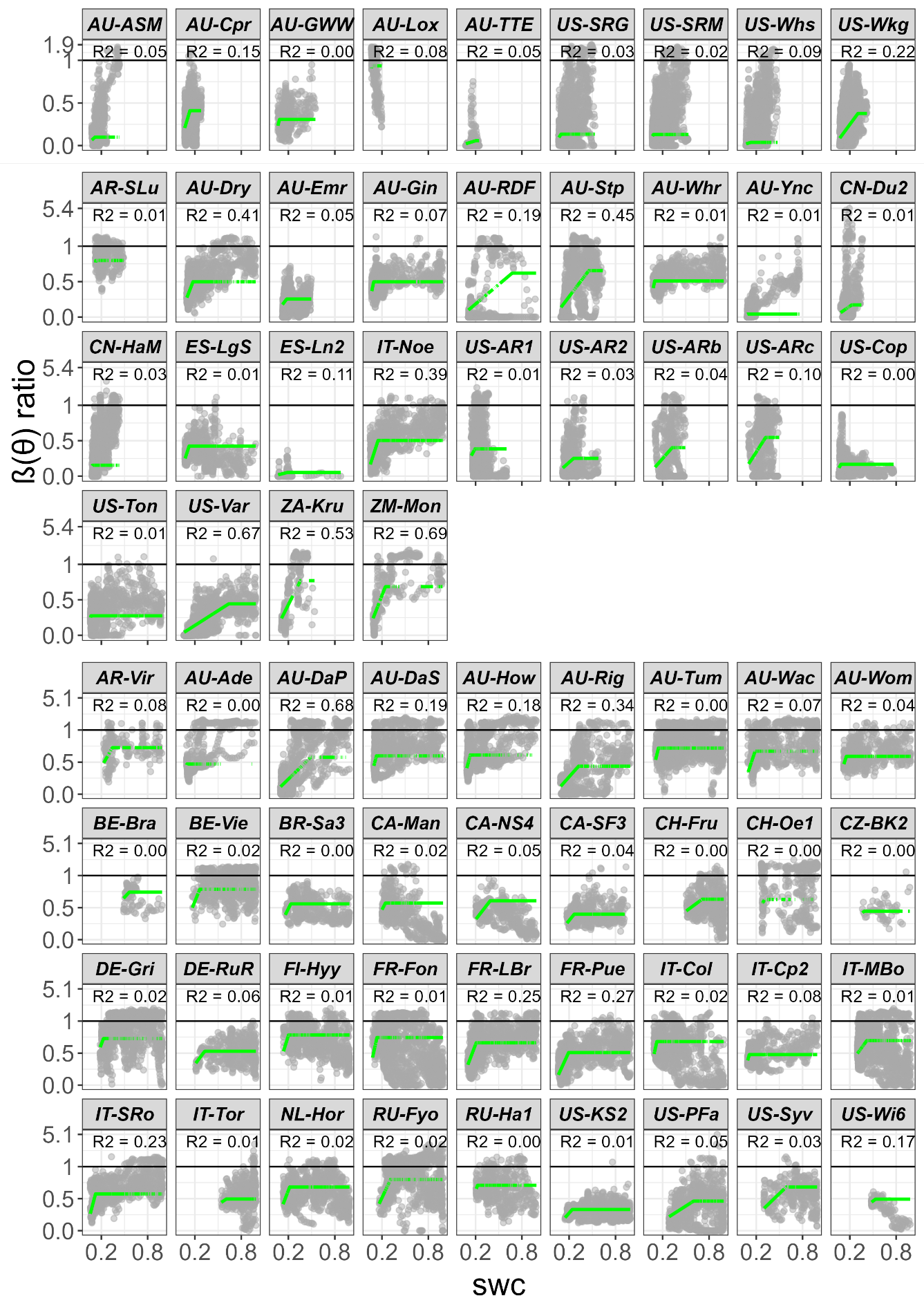


**Supplementary Figure 2**: Values of the fitted maximum β(θ) ratio (the ratio of actual flux-derived to modelled well-watered gross primary production) and the critical threshold value of soil moisture for all 67 sites used in the analysis, where the intercept is assumed to be zero. The βθ ratio and the soil water content (swc) are both unitless. Note that the scale above 1 has been compressed for visualization purposes.


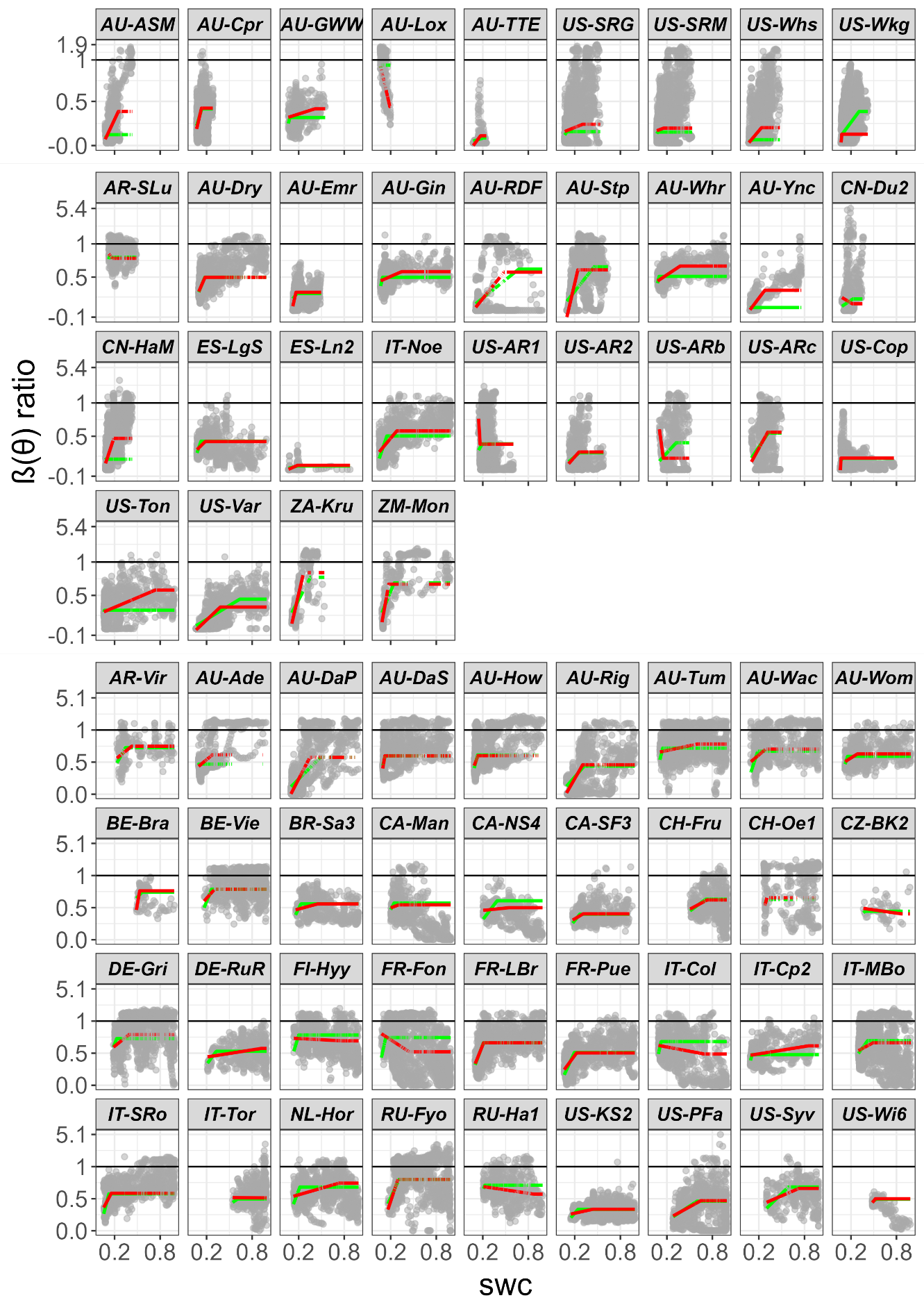


**Supplementary Figure 3**: Values of the fitted maximum β(θ) ratio (the ratio of actual flux-derived to modelled well-watered gross primary production) and the critical threshold value of soil moisture for all 67 sites used in the analysis, where the intercept is assumed to be zero (green line) or not fixed (red line). The βθ ratio and the soil water content (swc) are both unitless. Note that the scale above 1 has been compressed for visualization purposes.


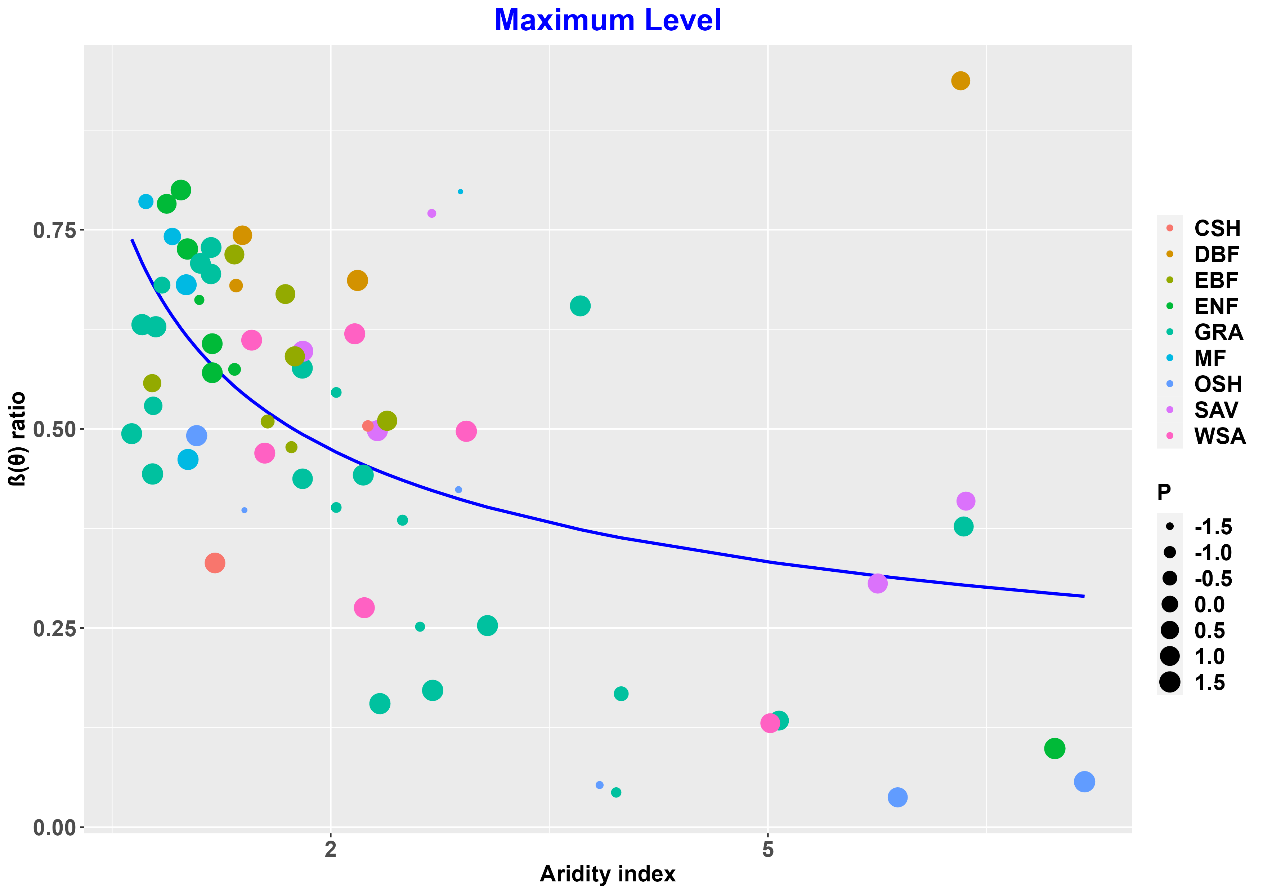


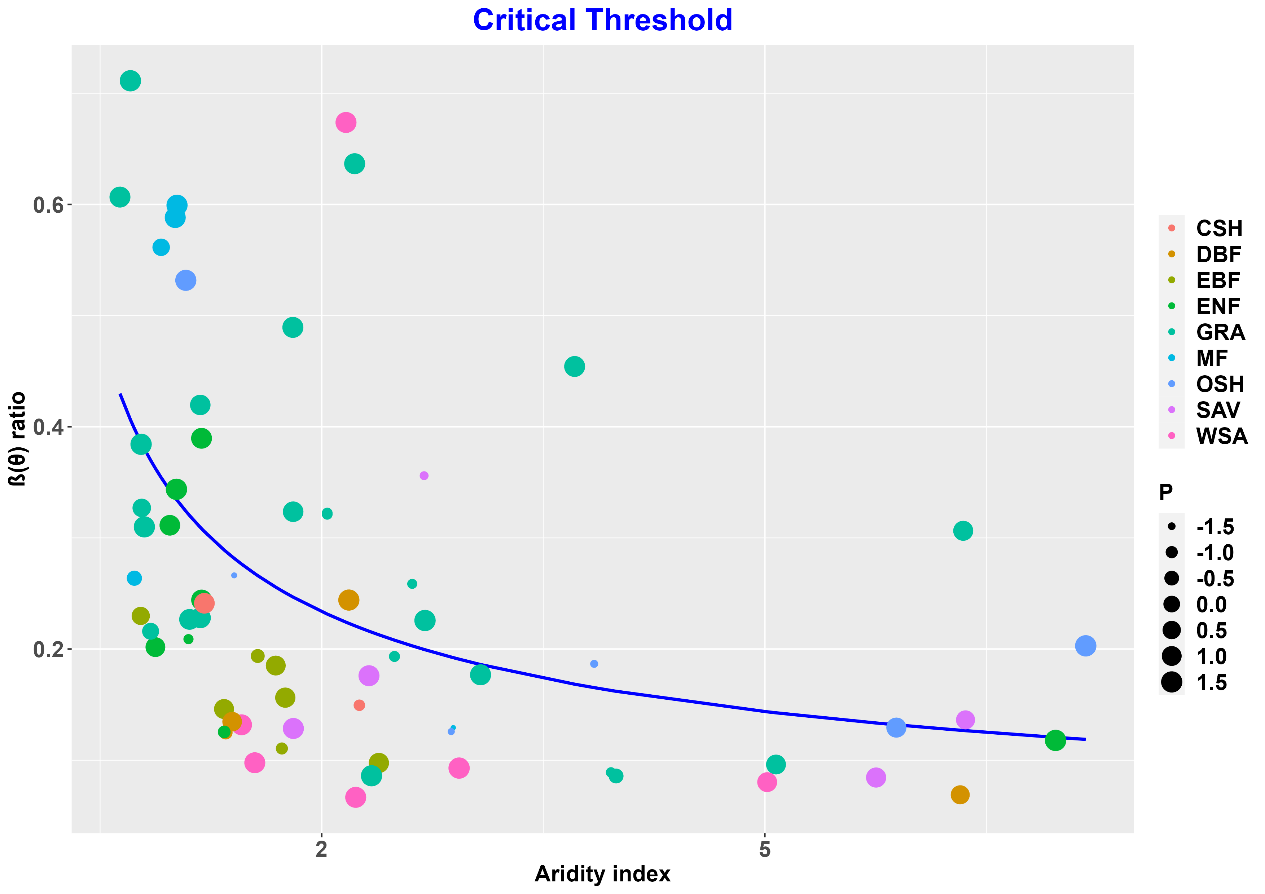


**Supplementary Figure 4**: The fitted non-linear regression model of the maximum level (top) and the critical threshold (bottom) of the β(θ) ratio (the ratio of observed to predicted gross primary production) against the aridity index, where the sites are classified according to vegetation type and precipitation phase.


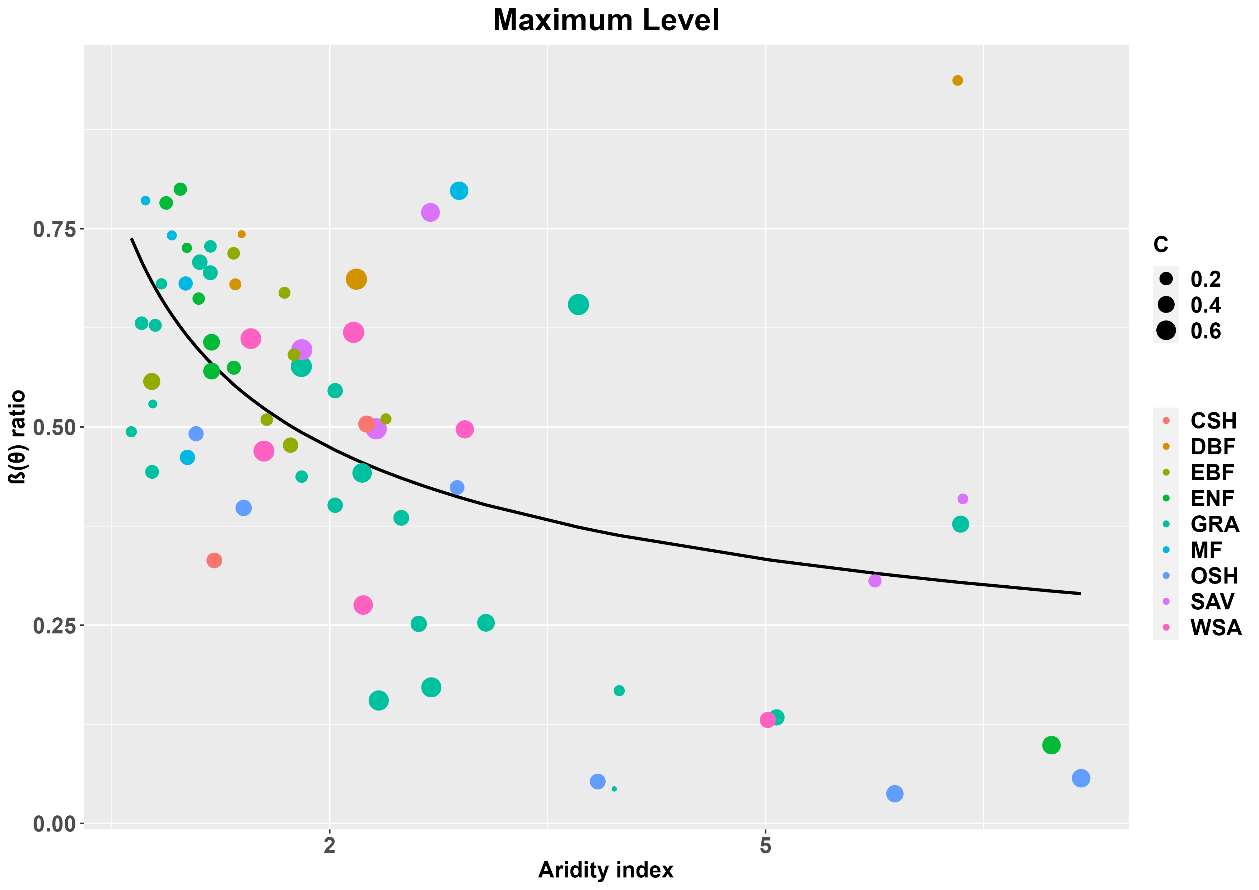


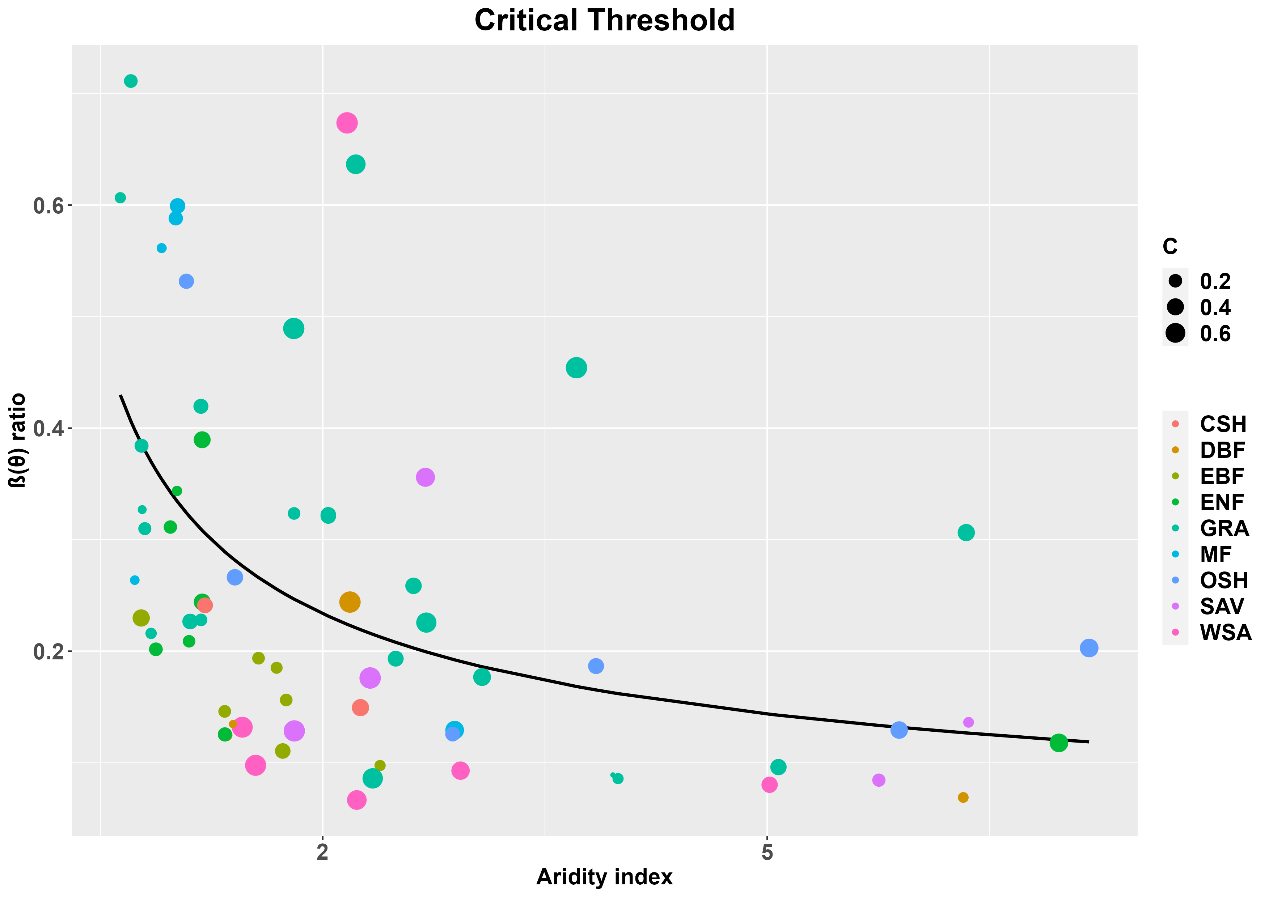


**Supplementary Figure 5**: The fitted non-linear regression model of the maximum level (top) and the critical threshold (bottom) of the β(θ) ratio (the ratio of observed to predicted gross primary production) against the aridity index, where the sites are classified according to according to vegetation type and precipitation concentration.


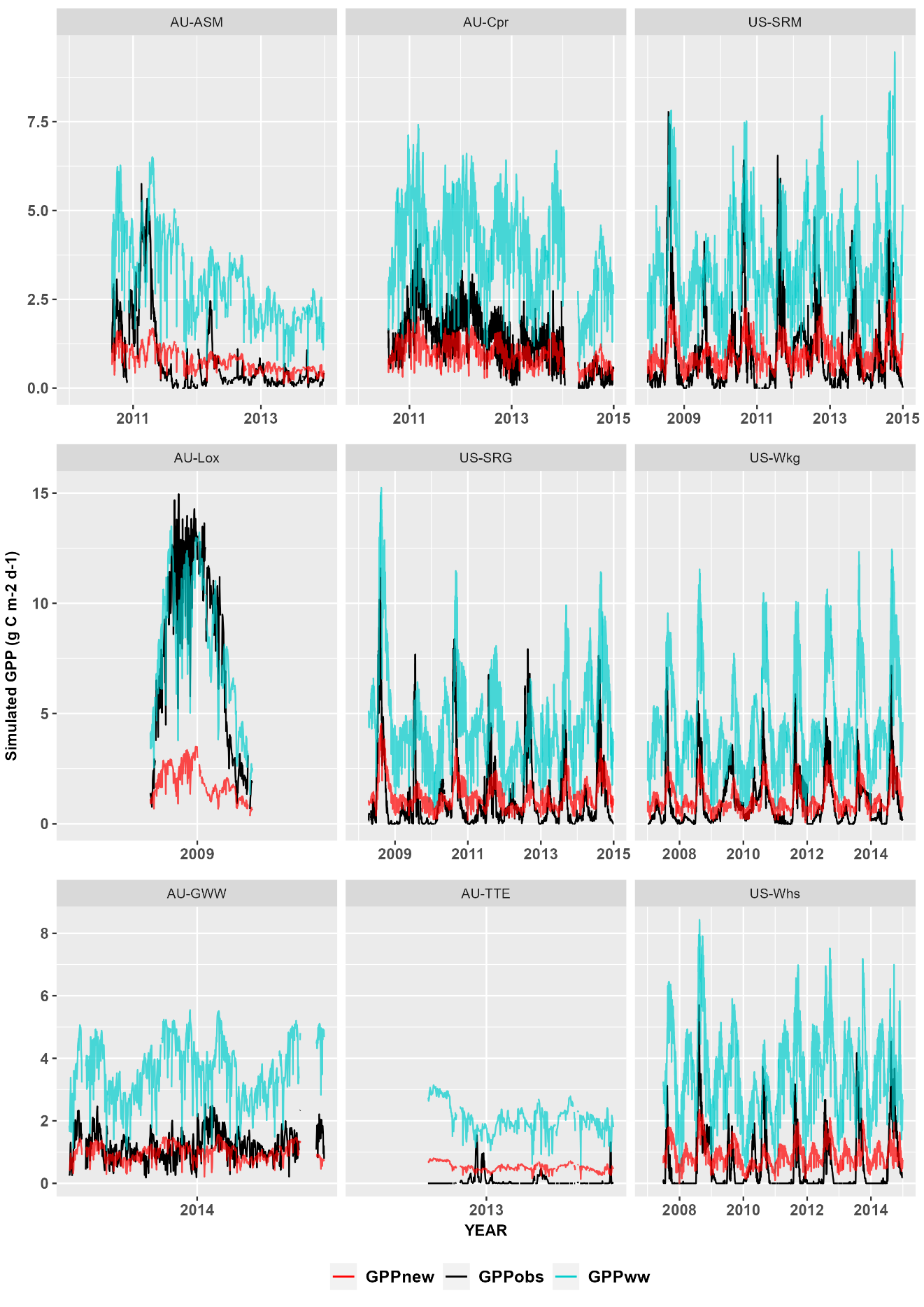


**Supplementary Figure 6**: The impact of the application of the new soil moisture stress function on simulated gross primary production (GPP_new_) at flux tower sites classified as arid (aridity index, AI >5). The new model is compared to the simulated level of GPP under well-watered conditions (GPP_ww_) and to flux-derived values (GPP_obs_).


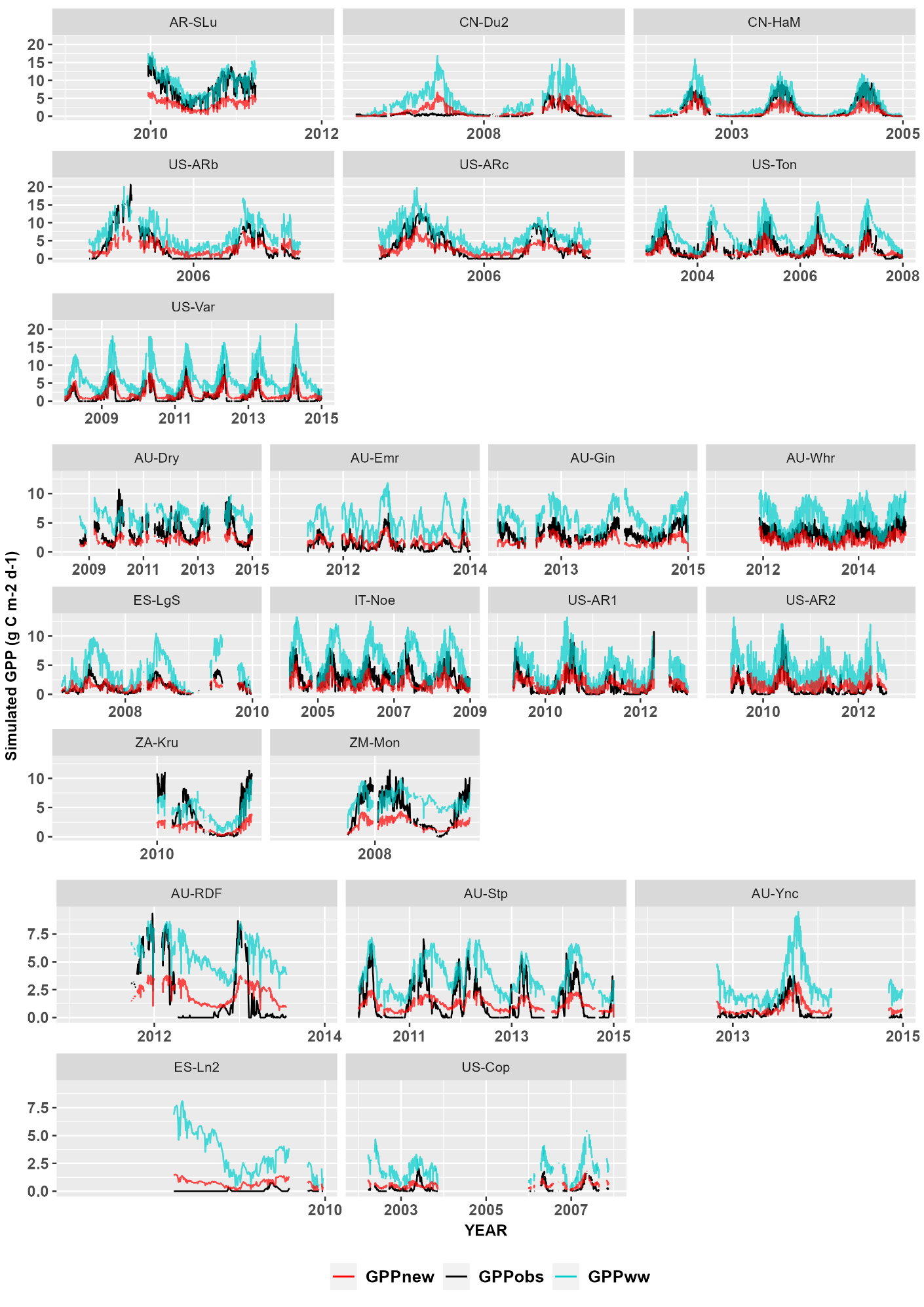


**Supplementary Figure 7**: The impact of the application of the new soil moisture stress function on simulated gross primary production (GPP_new_) at flux tower sites classified as semi-arid (aridity index, AI between 2 and 5). The new model is compared to the simulated level of GPP under well-watered conditions (GPP_ww_) and to flux-derived values (GPP_obs_).


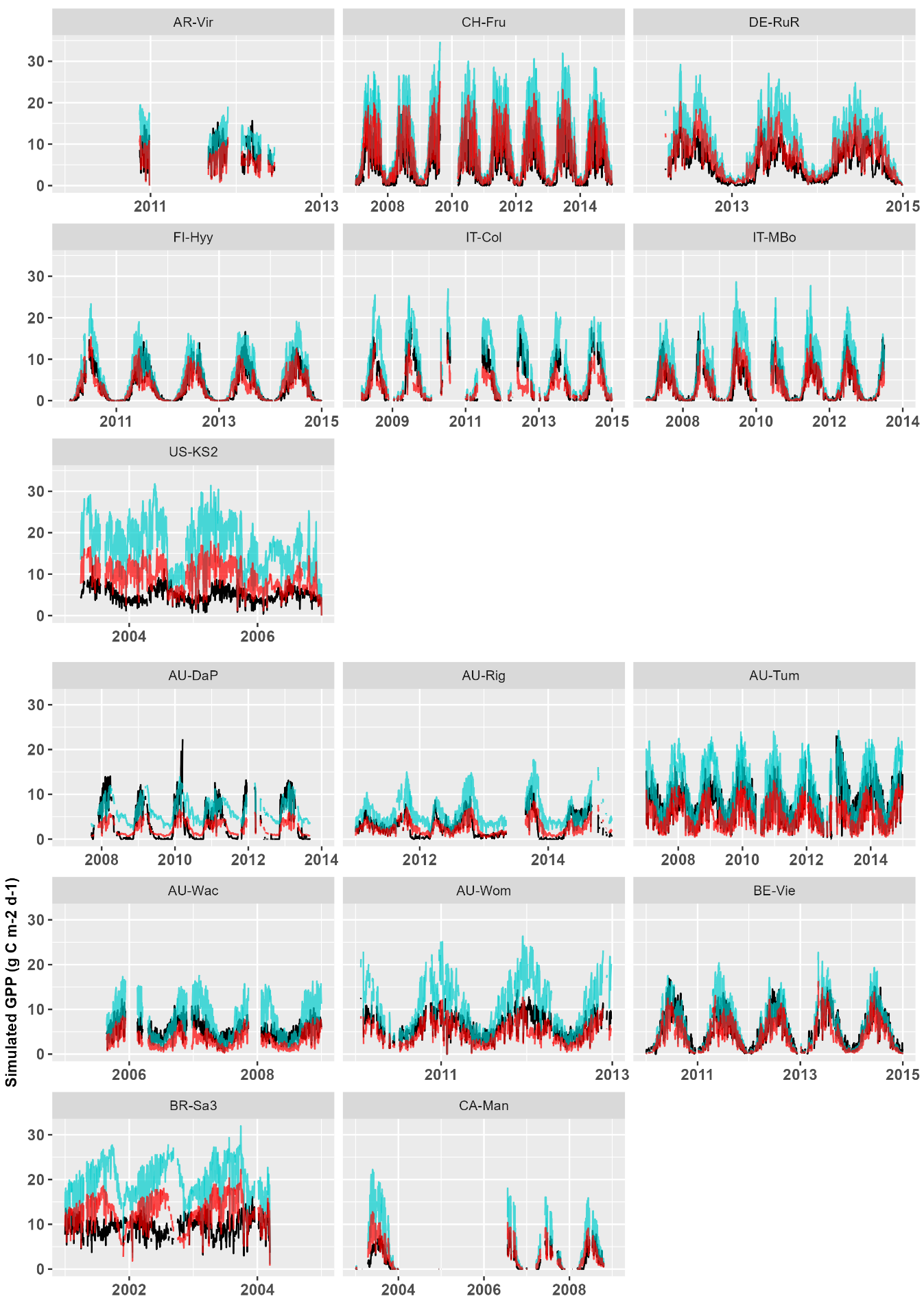


**Supplementary Figure 8**: The impact of the application of the new soil moisture stress function on simulated gross primary production (GPP_new_) at flux tower sites classified as humid (aridity index, AI <2). The new model is compared to the simulated level of GPP under well-watered conditions (GPP_ww_) and to flux-derived values (GPP_obs_).

Figure 8 (continued)


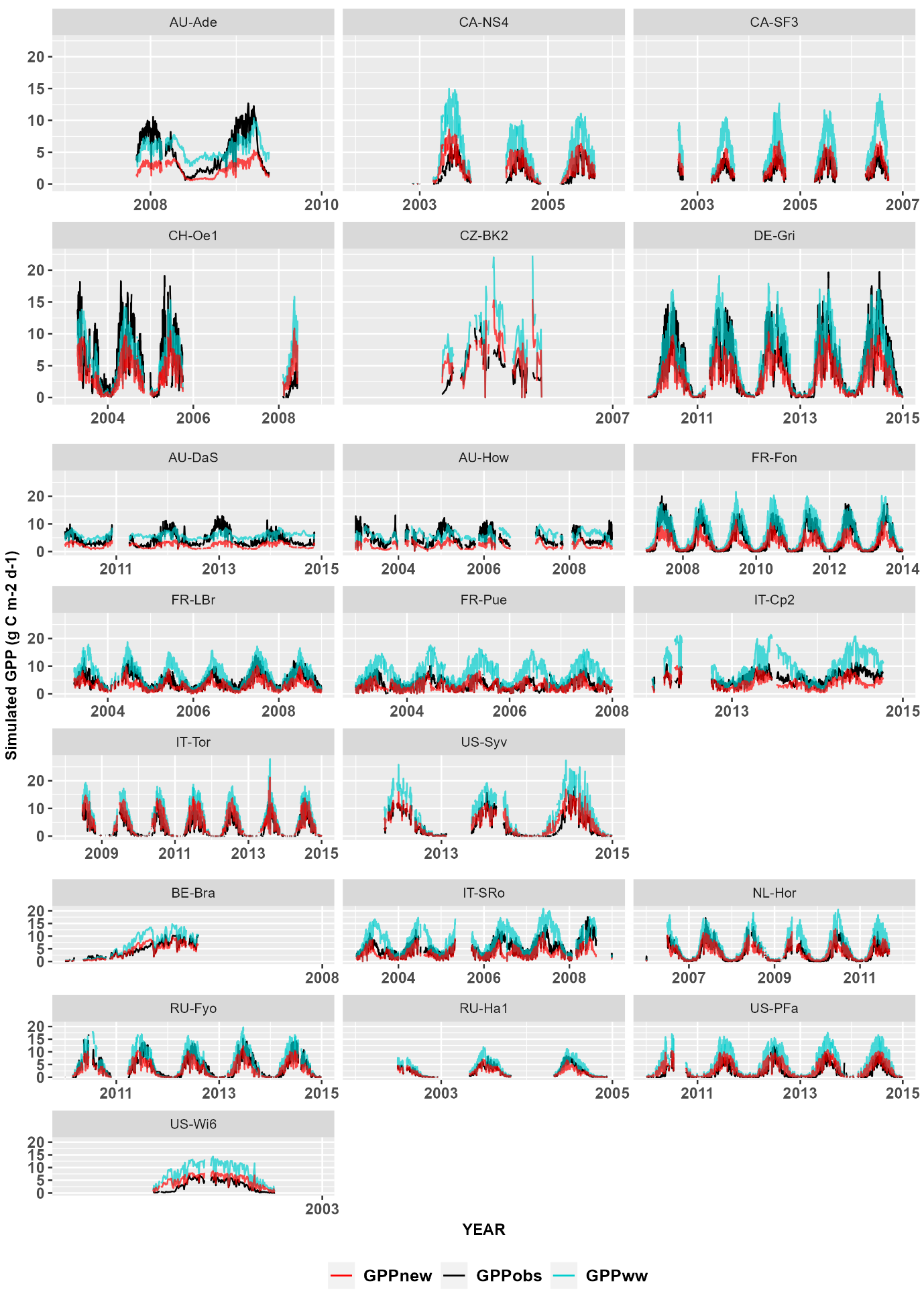


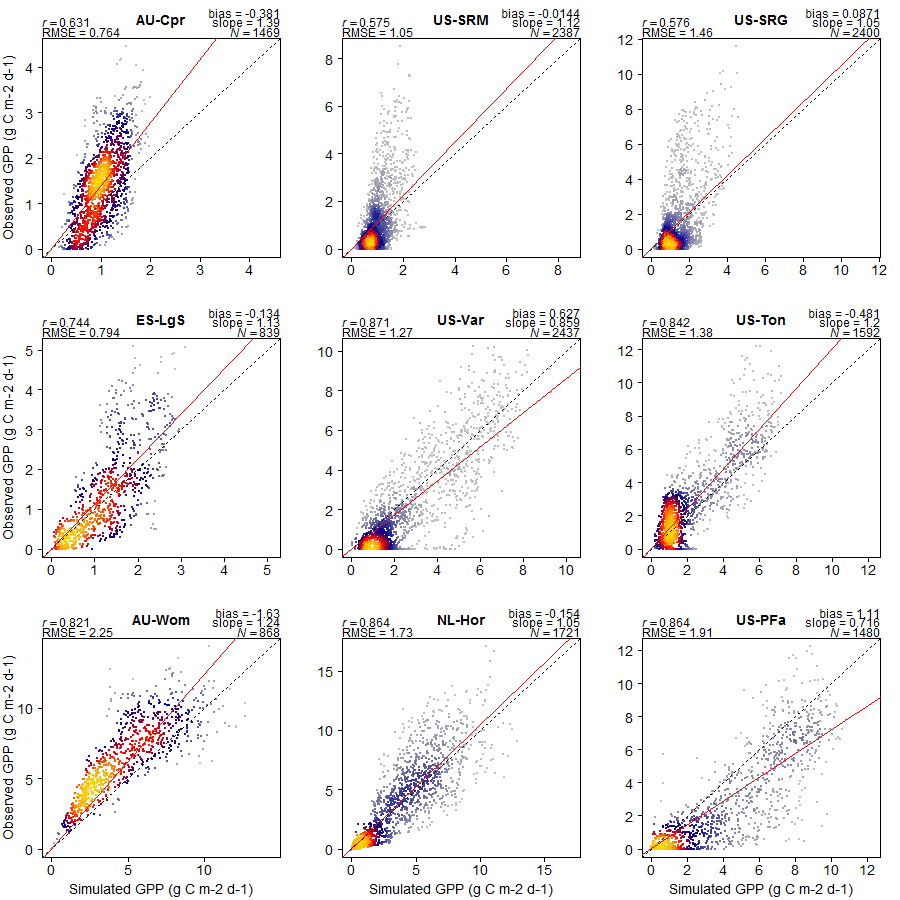


**Supplementary Figure 9**: Heat map plot comparing simulated and observed daily GPP across the same sites displayed in the Figure 6 in the main text. The scatter plot shows the gross primary production (GPP) simulated using the new soil-moisture stress function (GPPnew—Simulated GPP) at six flux tower sites representing the range of climatological aridity compared to the to flux-derived values (GPP_obs_ – Observed GPP). The colour scale indicated data density, with red areas representing the highest concentration of points. The 1:1 line represent the perfect agreement between observations and simulations.


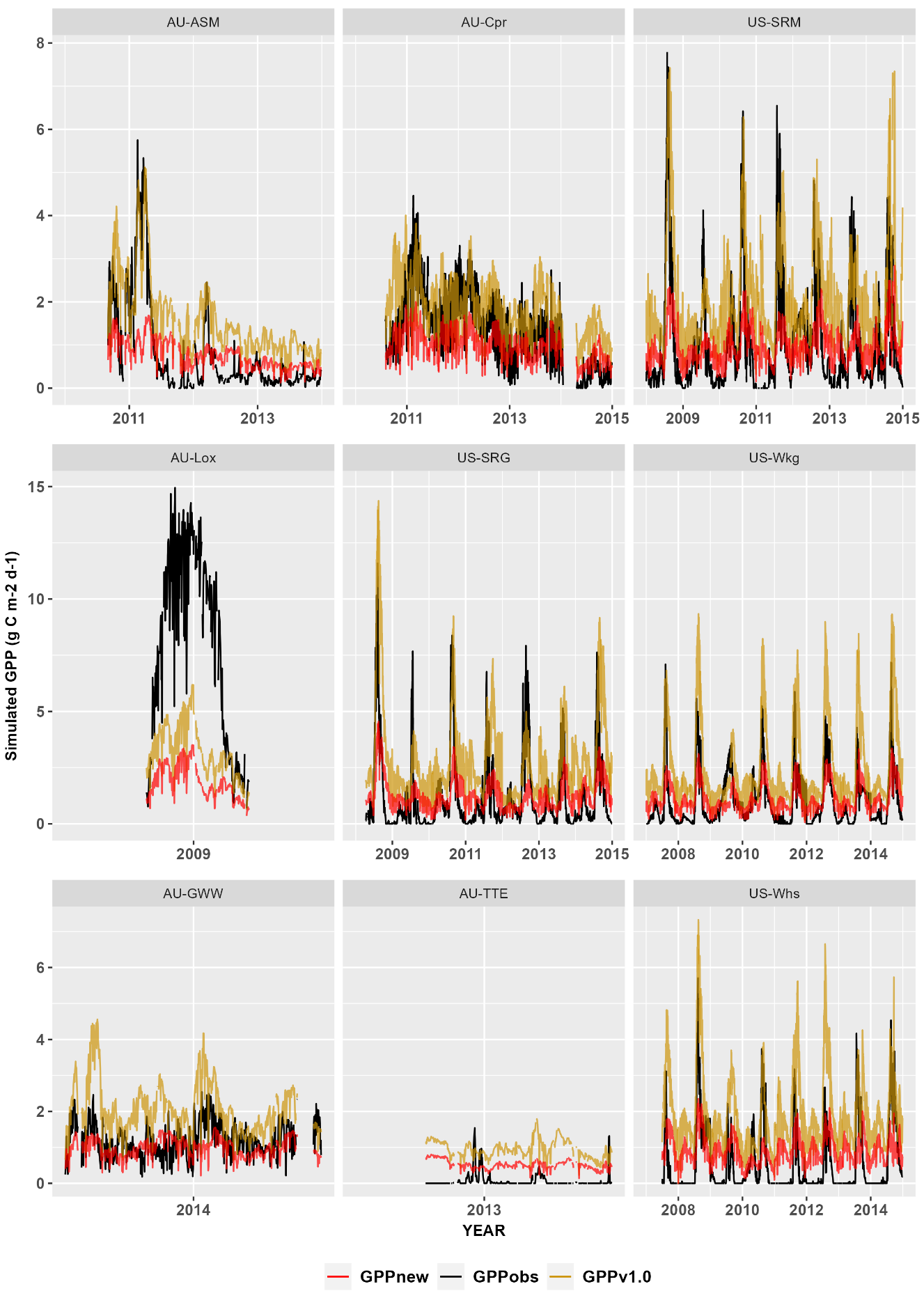


**Supplementary Figure 10**: Comparison of simulated gross primary production including the new soil-moisture stress function (GPP_new_) and the original stress function (GPP_v1.0_) from Stocker et al. (2020) against flux-derived values (GPP_obs_) at flux tower sites classified as arid (aridity index, AI > 5).


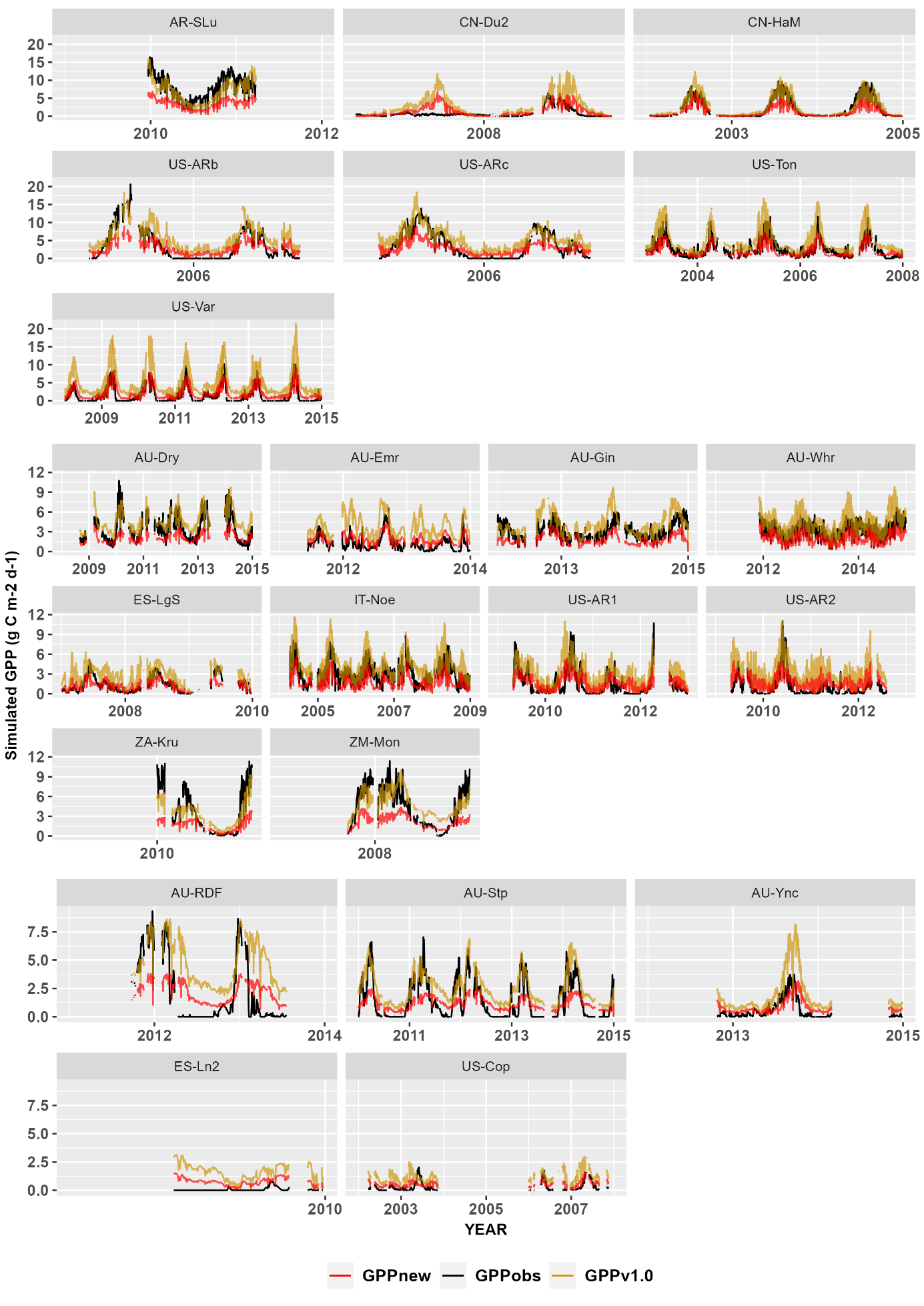


**Supplementary Figure 11**: Comparison of simulated gross primary production including the new soil-moisture stress function (GPP_new_) and the original stress function (GPP_v1.0_) from Stocker et al. (2020) against flux-derived values (GPP_obs_) at flux tower sites classified as semi-arid (aridity index, AI = between 2 and 5).


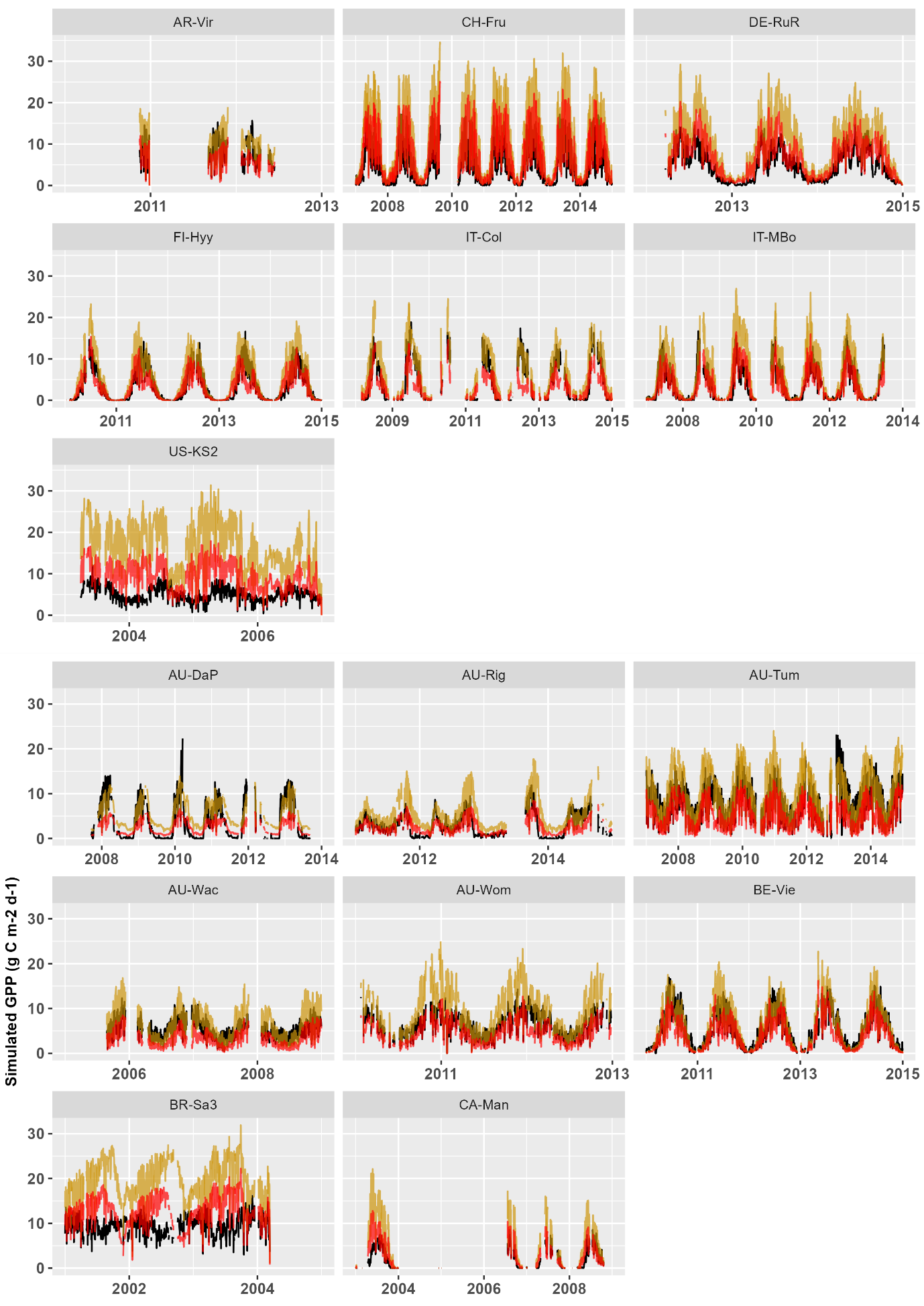


**Supplementary Figure 12**: Comparison of simulated gross primary production including the new soil-moisture stress function (GPP_new_) and the original stress function (GPP_v1.0_) from Stocker et al. (2020) against flux-derived values (GPP_obs_) at flux tower sites classified as humid (aridity index, AI < 2).

Figure 12 (continued)


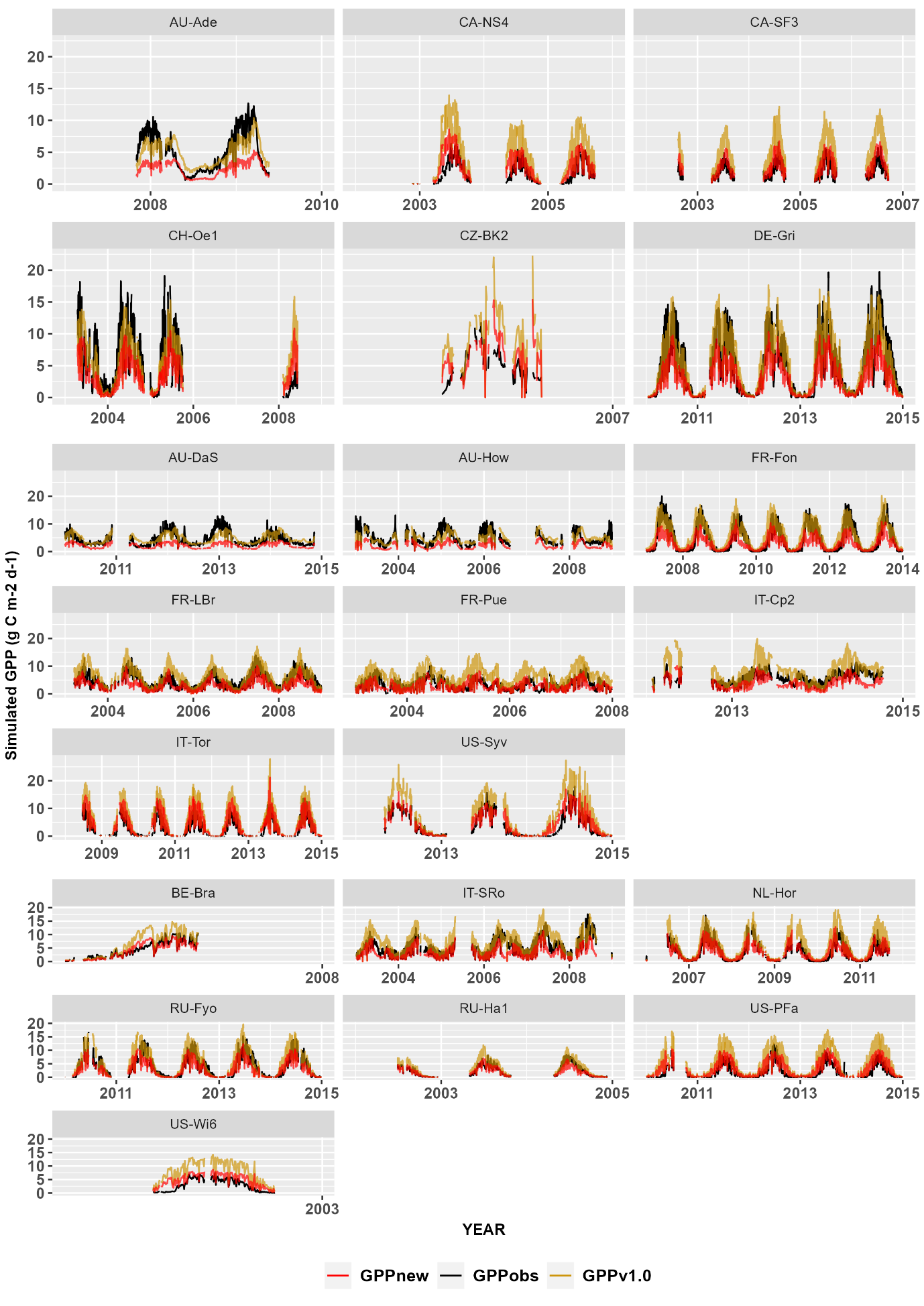


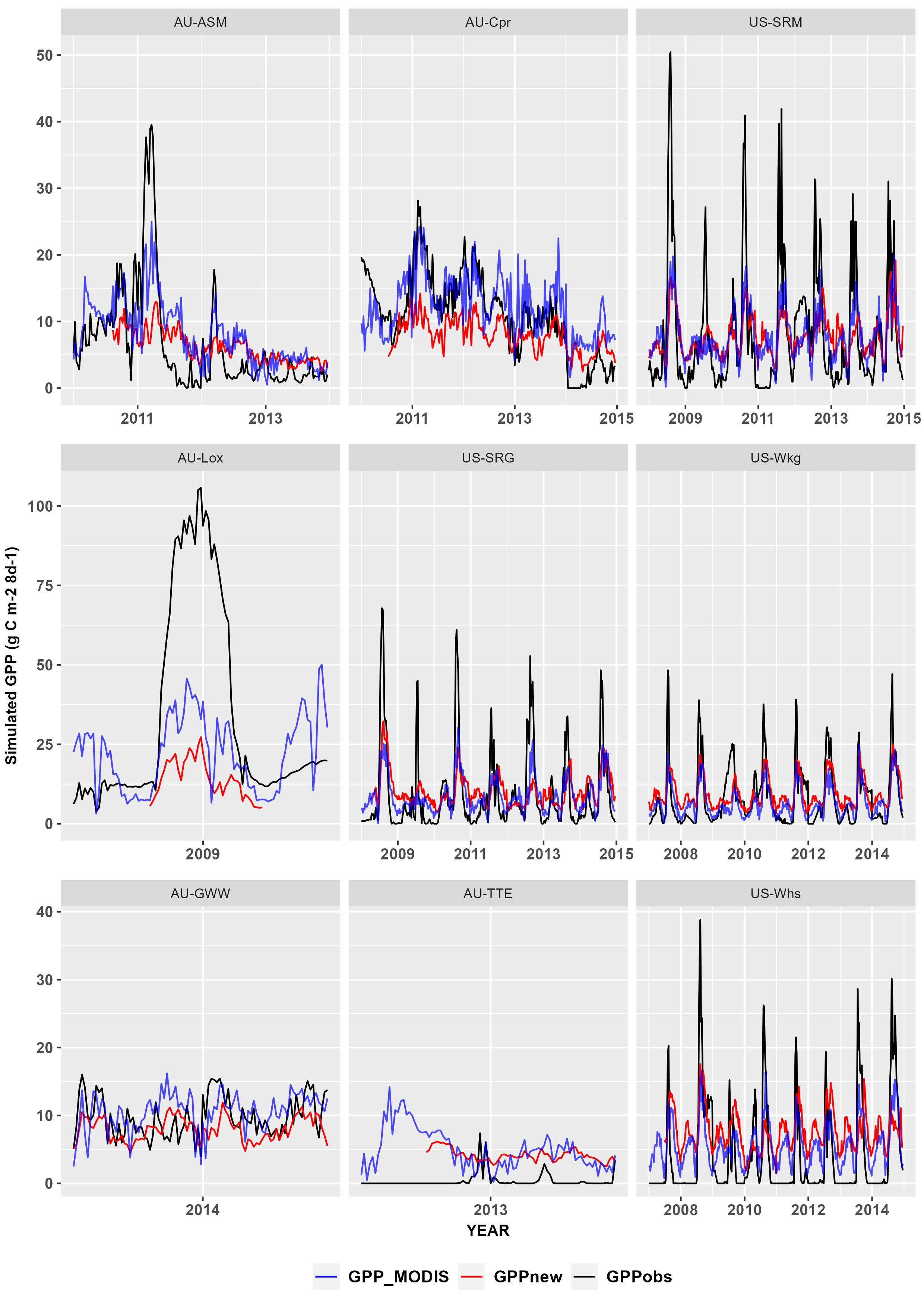


**Supplementary Figure 13**: Comparison of simulated gross primary production including the new soil-moisture stress function (GPPnew) and the gross primary production simulated by MOD17A2HGF v0.61 (GPP_MODIS_) against flux-derived values (GPPobs) at flux tower sites classified as arid (aridity index, AI > 5).


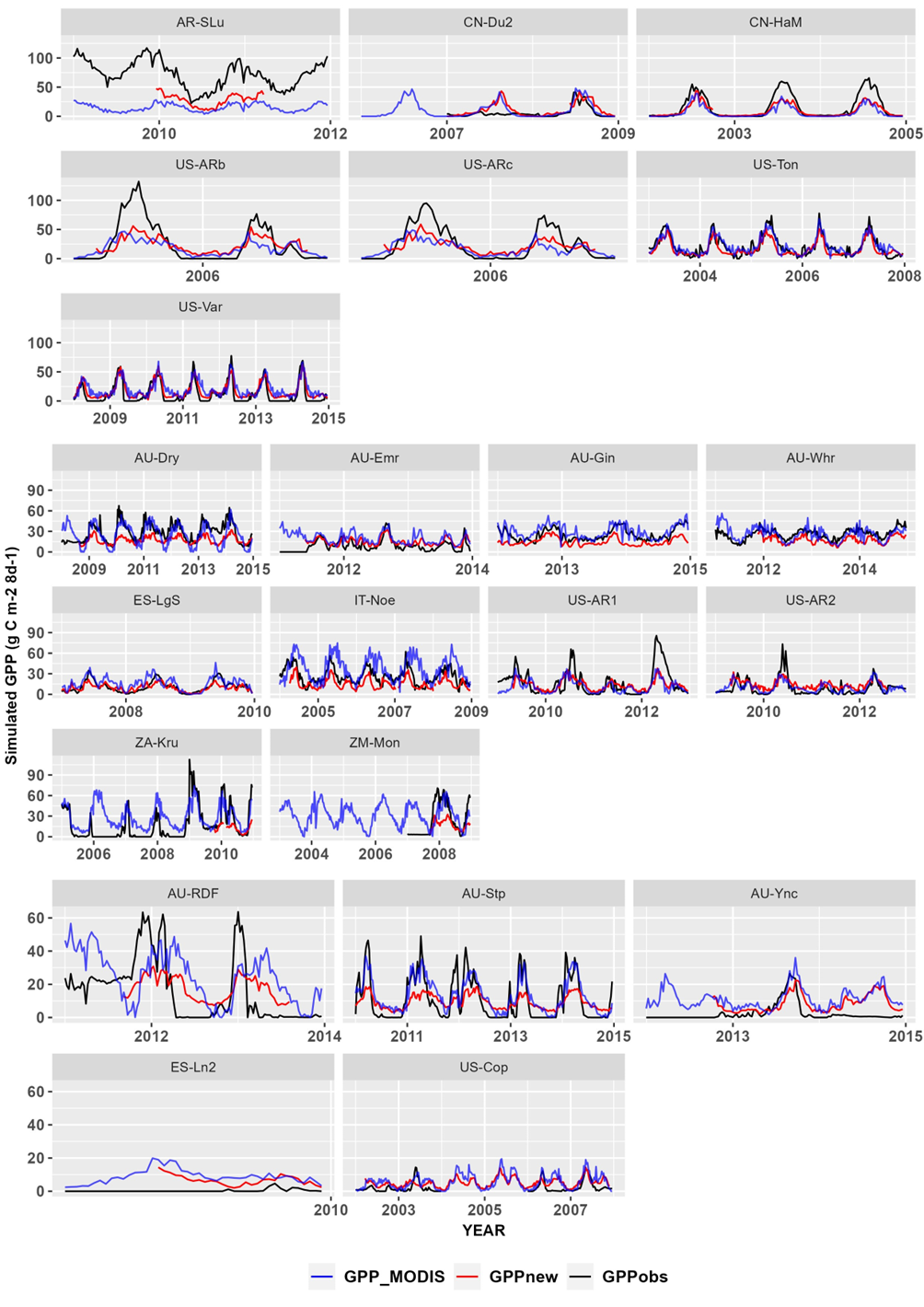


**Supplementary Figure 14**: Comparison of simulated gross primary production including the new soil-moisture stress function (GPPnew) and the gross primary production simulated by MOD17A2HGF v0.61 (GPP_MODIS_) against flux-derived values (GPPobs) at flux tower sites classified as semi-arid (aridity index, AI = between 2 and 5).


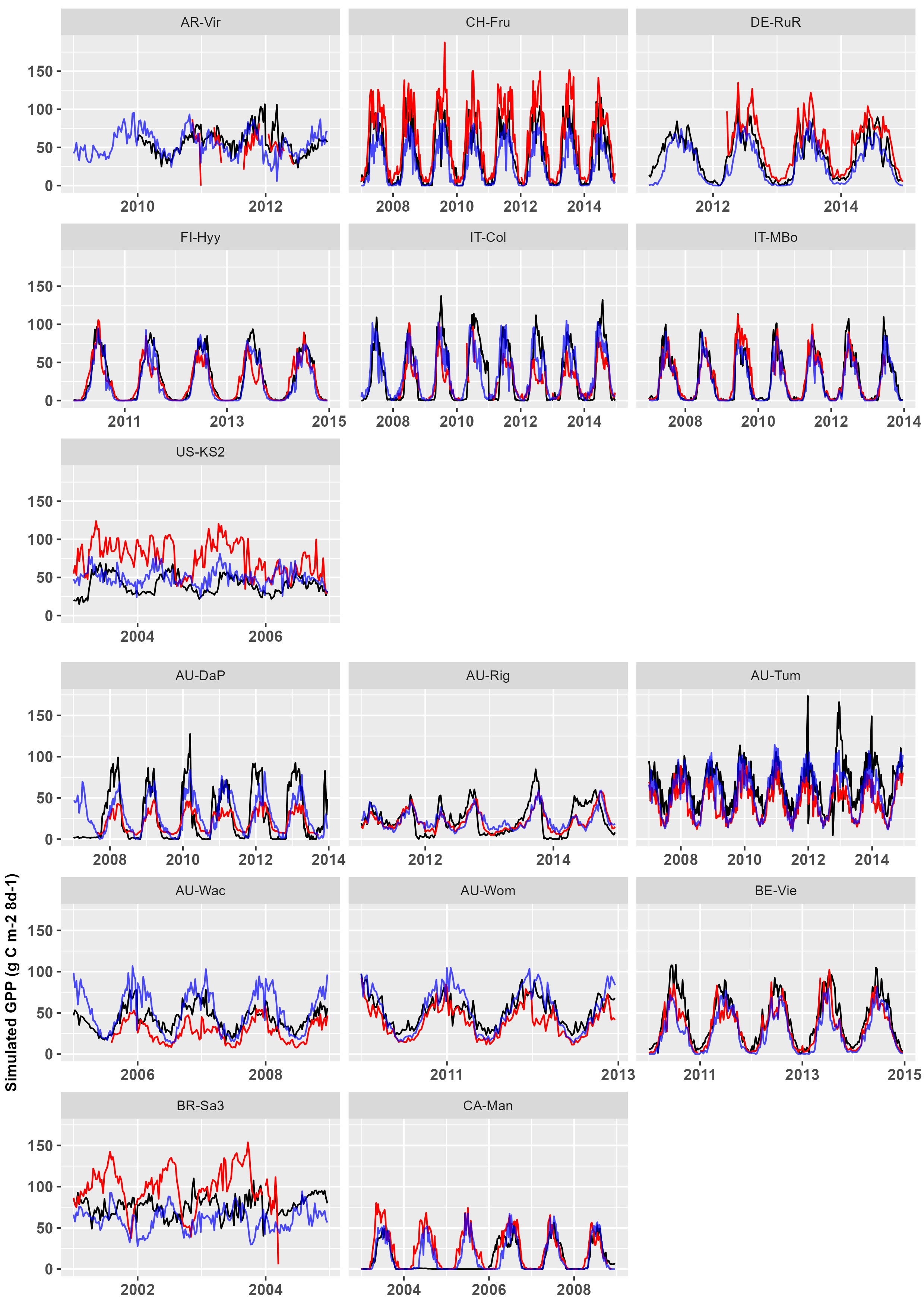


**Supplementary Figure 15**: Comparison of simulated gross primary production including the new soil-moisture stress function (GPPnew) and the gross primary production simulated by MOD17A2HGF v0.61 (GPP_MODIS_) against flux-derived values (GPPobs) at flux tower sites classified as humid (aridity index, AI < 2).

Figure 15 (continued)


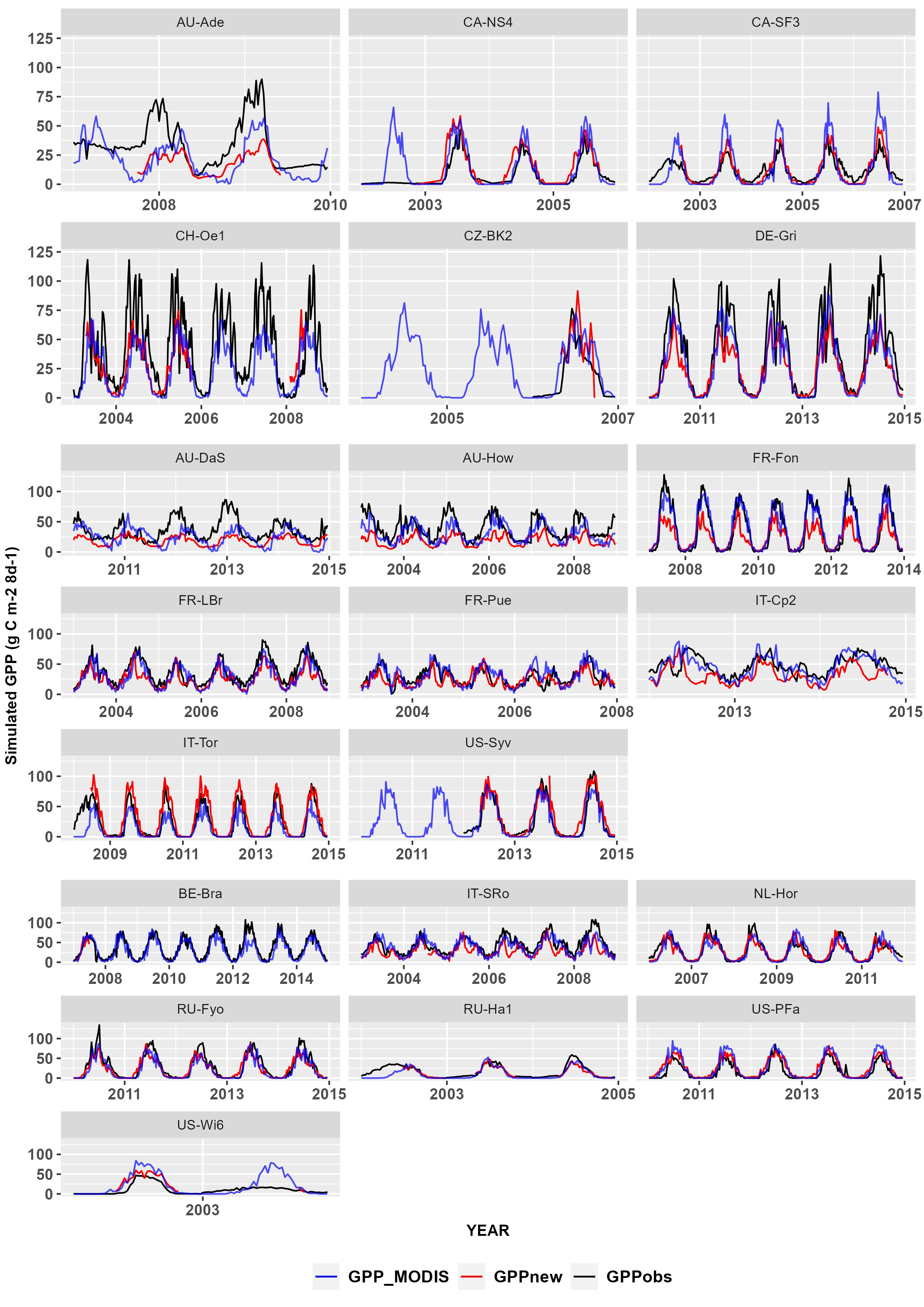


**
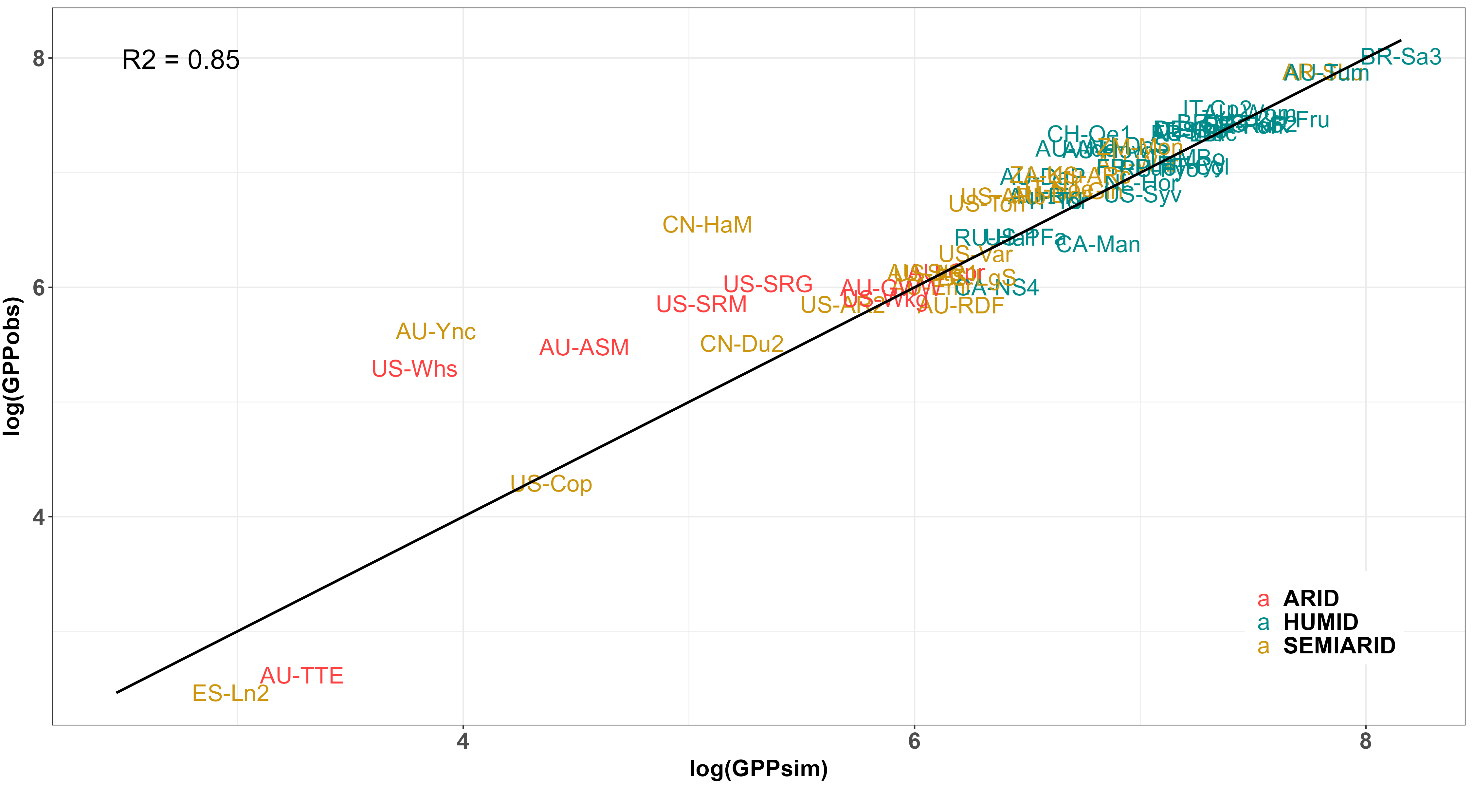
**

**Supplementary Figure 16**: Comparison of simulated total annual gross primary production using site-specific parameters and the flux-derived total annual gross primary production across 67 sites classified by aridity (arid, semi-arid, humid). Points represent log-transformed values of observed (log(GPPobs)) and simulated (log(GPPsim)) GPP at different sites. Each point represents the multi-year mean of total annual GPP at each site, calculated from years with >50% data coverage and >0.8 data quality. The 1:1 line indicates ideal model performance. GPP is in units of gC m−2 year−1. The r² value (0.85) indicates slightly improved model performance using site-specific parameters compared to the aridity-based approach presented in Figure 8."

**Supplementary text**: Potential explanations for the poor model performance at ES-Ln2 and AU-TTE.

The application of the new function results in an overall improvement in model performance, although there is a tendency to underestimate GPP slightly in semi-arid and humid sites. There is no clear systematic bias across aridity classes, and a good fit with the observations is obtained for the majority of sites analysed. However, there are two sites where the model fails to match observed GPP (Figure 8; Supplementary Figures 6, 7):

1. **Lanjaron-Salvage logging (ES-Ln2)** – This site, an "Open shrubland" according to FLUXNET’s International Geosphere-Biosphere Programme (IGBP) classification, is actually a managed pine plantation where a fire occurred in 2005. Post-fire treatments of "salvage logging" and "no intervention" were applied, with measurements in 2009 (i.e. the year we analysed here) examining the impact of these treatments (Serrano-Ortiz et al., 2011). The site's unique management history may explain its exclusion from the PLUMBER dataset (<https://researchdata.edu.au/plumber2-forcing-evaluation-surface-models/1656048>). It suggests that the observed discrepancy between simulated and observed GPP reflects site-specific factors rather than model inaccuracy.
2. **Ti Tree East (AU-TTE)** – This site is classified as grassland with Mulga woodland, but is located in the Pine Hill cattle station and was grazed up to three years before the flux tower was set up. The site experiences high seasonal (monsoonal) rainfall concentrated during the austral summer months (on average 72% of rainfall falls during December, January and February; 86% of median rainfall occurs from November to April (Cleverly et al., 2013; Cleverly et al., 2016a; Cleverly et al., 2016b). However, annual rainfall as recorded at the nearby Bureau of Meteorology station on the Territory Grape Farm (<http://www.bom.gov.au>) varies significantly, with 100 mm in 2009, 750 mm in 2010, 422 mm in 2016, and 108 mm in 2018. In 2012 and 2013, the years analysed in this study, rainfall was stable (~200-250 mm), but the variability before and after these years indicates it is inappropriate to consider this an arid site. The discrepancy between simulated and observed GPP is likely to be a consequence of this rainfall variability.

**References**

Cleverly, J., Boulain, N., Villalobos-Vega, R., Grant, N., Faux, R., Wood, C., Cook, P. G., Yu, Q., Leigh, A., & Eamus, D.: Dynamics of component carbon fluxes in a semi-arid Acacia woodland, central Australia. *Journal of Geophysical Research: Biogeosciences*, **118**(3), 1168–1185. <https://doi.org/10.1002/jgrg.20101>, 2013.

Cleverly, J., Eamus, D., Restrepo Coupe, N., Chen, C., Maes, W., Li, L., Faux, R., Santini, N. S., Rumman, R., Yu, Q., & Huete, A.: Soil moisture controls on phenology and productivity in a semi-arid critical zone. *Science of The Total Environment*, **568**, 1227–1237. <https://doi.org/10.1016/j.scitotenv.2016.05.142>, 2016a.

Cleverly, J., Eamus, D., Van Gorsel, E., Chen, C., Rumman, R., Luo, Q., Coupe, N. R., Li, L., Kljun, N., Faux, R., Yu, Q., & Huete, A.: Productivity and evapotranspiration of two contrasting semiarid ecosystems following the 2011 global carbon land sink anomaly. *Agricultural and Forest Meteorology*, **220**, 151–159. <https://doi.org/10.1016/j.agrformet.2016.01.086>, 2016b.

Serrano-Ortiz, P., Marañón-Jiménez, S., Reverter, B. R., Sánchez-Cañete, E. P., Castro, J., Zamora, R., & Kowalski, A. S.: Post-fire salvage logging reduces carbon sequestration in Mediterranean coniferous forest. *Forest Ecology and Management*, **262**(12), 2287–2296. <https://doi.org/10.1016/j.foreco.2011.08.023>, 2011.
